# Conditional Cell-Penetrating Peptide Exposure as Selective Nanoparticle Uptake Signal

**DOI:** 10.1021/acsami.4c07821

**Published:** 2024-07-16

**Authors:** Melanie Walter, Merlin Bresinsky, Oliver Zimmer, Steffen Pockes, Achim Goepferich

**Affiliations:** † Department of Pharmaceutical Technology, University of Regensburg, 93053 Regensburg, Bavaria, Germany; ‡ Department of Medicinal Chemistry I, University of Regensburg, 93053 Regensburg, Bavaria, Germany

**Keywords:** nanoparticle targeting, polymer nanoparticles, polyarginine, TAT, nanoparticle surface charge, polycationic, charge-mediated uptake, sequential uptake

## Abstract

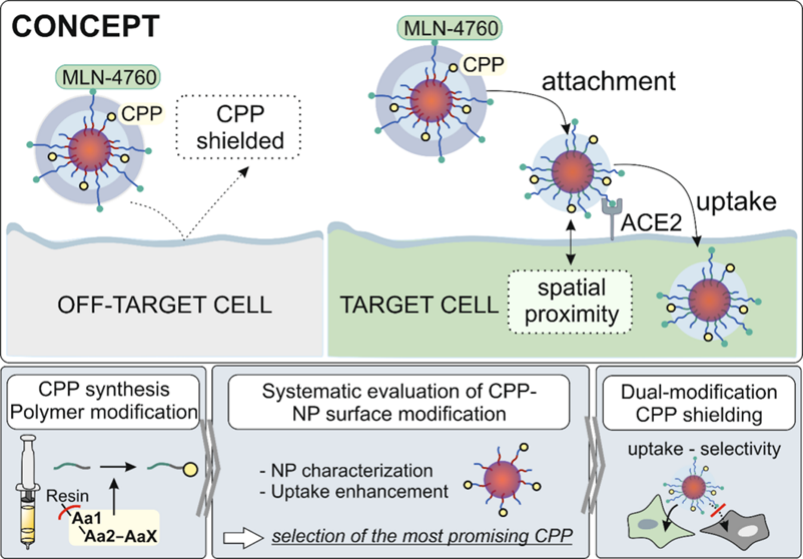

A major bottleneck diminishing the therapeutic efficacy of various drugs is that only small proportions of the administered dose reach the site of action. One promising approach to increase the drug amount in the target tissue is the delivery via nanoparticles (NPs) modified with ligands of cell surface receptors for the selective identification of target cells. However, since receptor binding can unintentionally trigger intracellular signaling cascades, our objective was to develop a receptor-independent way of NP uptake. Cell-penetrating peptides (CPPs) are an attractive tool since they allow efficient cell membrane crossing. So far, their applicability is severely limited as their uptake-promoting ability is nonspecific. Therefore, we aimed to achieve a conditional CPP-mediated NP internalization exclusively into target cells. We synthesized different CPP candidates and investigated their influence on nanoparticle stability, ζ-potential, and uptake characteristics in a core–shell nanoparticle system consisting of poly(lactid-*co*-glycolid) (PLGA) and poly(lactic acid)-poly(ethylene glycol) (PLA_10k_PEG_2k_) block copolymers with CPPs attached to the PEG part. We identified TAT47–57 (TAT) as the most promising candidate and subsequently combined the TAT-modified PLA_10k_PEG_2k_ polymer with longer PLA_10k_PEG_5k_ polymer chains, modified with the potent angiotensin-converting enzyme 2 (ACE2) inhibitor MLN-4760. While MLN-4760 enables selective target cell identification, the additional PEG length hides the CPP during a first unspecific cell contact. Only after the previous selective binding of MLN-4760 to ACE2, the established spatial proximity exposes the CPP, triggering cell uptake. We found an 18-fold uptake improvement in ACE2-positive cells compared to unmodified particles. In summary, our work paves the way for a conditional and thus highly selective receptor-independent nanoparticle uptake, which is beneficial in terms of avoiding side effects.

## 1 Introduction

**Figure 1:**
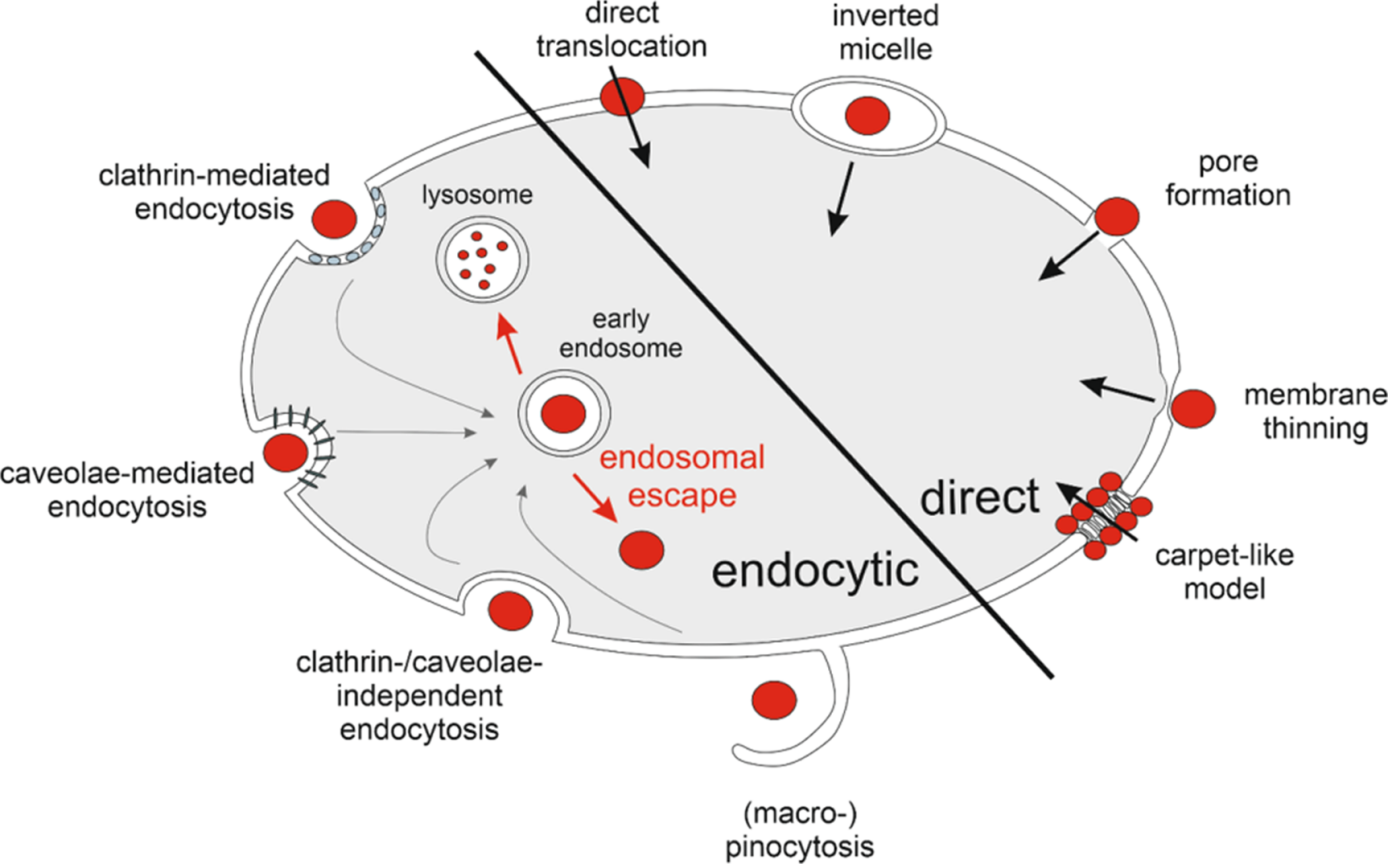
Overview of uptake mechanisms of cell-penetrating peptides. The uptake mechanism can be categorized into endocytic (left) and direct (right) uptake ways. While the compound is instantly available in the cytoplasm after direct uptake, it initially ends up in early endosomes after endocytic uptake.^12,13^ In this case, a further hurdle must be circumvented by means of endosomal escape in order to enter the cytoplasm. If this is not possible, the early endosomes undergo a maturing process, ending up in lysosomes, where acidic pH values and lysosomal enzymes lead to the degradation of the cargo.^14^

Cell-penetrating peptides (CPPs) are molecules of 4–40 mostly cationic amino acids and possess unique abilities for crossing biological barriers, including cell membranes.^1,2^ Therefore, they find broad applications to promote the cell internalization of various cargos.^3−6^ Their membrane-penetrating properties not only enable direct cell uptake via multiple pathways but are also considered to support endosomal escape (Figure 1).^7,8^ Thus, CPPs are a promising delivery system for drugs with intracellular targets and are attracting great interest as a tool for nanoparticle (NP) surface modification.^9−11^ However, the application of CPPs in the case of selective nanotherapy comes with severe challenges.

Although the mechanisms of CPP uptake have been studied intensively, they are not yet fully understood.^15,16^ The process and extent of CPP-cargo-conjugate internalization and their endosomal escape depend on various factors like cargo size, hydrophobicity, CPP concentration and surface density, and the resulting net charge.^13,15,17,18^ There is also a great variability among CPPs, which can be categorized into different groups: cationic, amphipathic, and hydrophobic CPPs. These groups have highly different properties, which in turn affect the CPP-cargo-conjugate characteristics.^6,15,19^ Moreover, there is also a lack of comprehensive systematic studies regarding nanoparticle surface modifications with CPPs that would facilitate an assessment of resulting particle properties and cell uptake characteristics. Finally, CPPs are typically nonselective and enhance nanoparticle uptake in nearly all types of cells,^20−22^ which is detrimental to target cell selectivity. Therefore, in the case of active nanoparticle targeting, where selective ligands are attached to the nanoparticle surfaces to identify a certain cell type or tissue, their application as a tool to trigger cell uptake is hardly feasible.^3,21^

**Figure 2:**
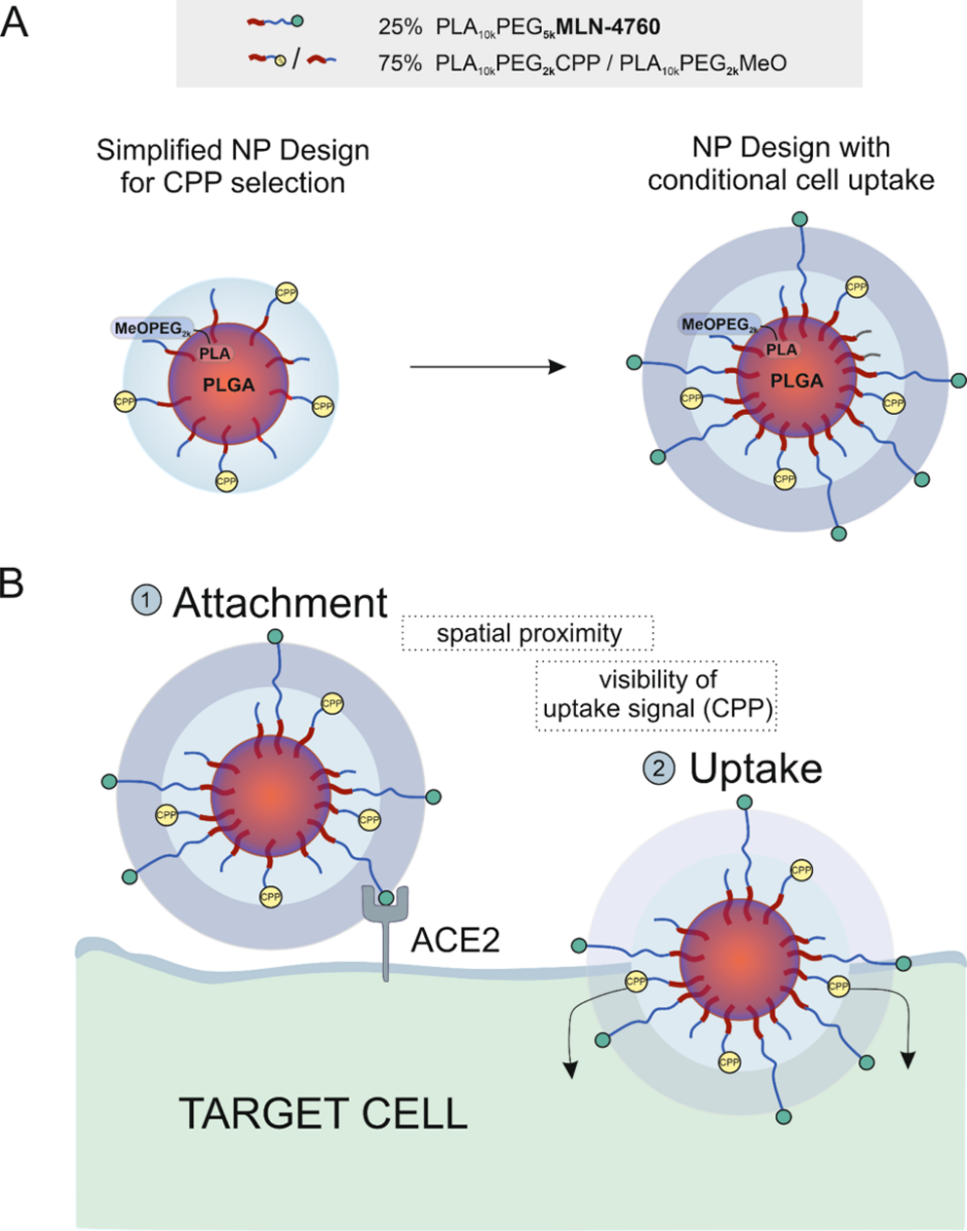
Nanoparticle structure and targeting approach. (A) Nanoparticle design. Different CPP modifications of polymeric nanoparticles with a core–shell structure were analyzed in a simplified particle design to select a suitable candidate for a conditional, sequential particle uptake (left). The nanoparticles consisted of PLGA and PLA_10k_PEG_2k_ block copolymers (PLA drawn in red, PEG in blue). Due to its higher hydrophilicity, PEG formed the NP shell (blue halo). Since PLGA and PLA are generally miscible,^26^ a PLGA-rich inner core layer (shown in orange/red) and a PLA-rich outer core layer (shown in red/blue) were obtained. The CPP was tethered to the PEG part and thus directly visible on the nanoparticle surface and able to mediate nanoparticle uptake. Methoxy-terminated polymers (PLA_10k_PEG_2k_MeO) served as space-filling polymers between the CPP-modified chains (PLA_10k_PEG_2k_CPP). The most promising candidate was established in a more complex particle design which promoted conditional, sequential nanoparticle uptake (right). Therefore, the CPP tethered to PLA_10k_PEG_2k_ was combined with PLA_10k_PEG_5k_ polymers, which had a longer PEG chain. The additional PEG length shielded the CPP during a first cell contact. Additionally, MLN-4760, a selective ACE2 inhibitor was attached to these longer polymer chains for selective target cell recognition (PLA_10k_PEG_5k_MLN). The proportion of long polymers was set to 25% according to Walter et al.^24^ For control nanoparticles, the MLN-modified polymer was replaced by uncharged methoxy-terminated polymer (PLA_10k_PEG_5k_MeO). Different amounts of CPP modifications were evaluated (0–75%) (PLA_10k_PEG_2k_CPP) and the proportion of short polymer, which should not be modified, was accordingly replaced by short methoxy-terminated polymers as placeholders (PLA_10k_PEG_2k_MeO). (B) Concept of a conditional, sequential nanoparticle uptake. Since the CPP is shielded by longer polymers during a first cell contact, the uptake-enhancing abilities of the ligand do not directly promote cell uptake. Only after a previous, selective binding of the nanoparticle to ACE2 via MLN-4760, the established spatial proximity exposes the uptake signal previously hidden inside the polymer shell. This leads to CPP-mediated uptake exclusively into ACE2-positive target cells.

In this work, we aimed to develop nanoparticles that rely on the cell uptake-enhancing properties of CPPs with utmost target cell selectivity. First, we systematically investigated nanoparticle modifications with various cell-penetrating peptides attached to the NP surface to identify the most suitable CPP candidate in terms of resulting particle size and stability. The nanoparticles had a core–shell structure, consisting of poly(d,l-lactide-*co*-glycolide) (PLGA) and a block copolymer of poly(lactic acid)-poly(ethylene glycol) (PLA_10k_PEG_2k_). The CPPs were tethered to the PEG part, which forms the nanoparticle shell, and were therefore localized directly visible on the particle surface (Figure 2A, left). After selection of the most appropriate CPP, the CPP-modified polymer was combined with PLA_10k_PEG_5k_ polymer chains. On the one hand, the longer PEG chains hide the CPP inside the PEG shell and thus prevent unspecific cell internalization upon a first cell contact. On the other hand, PLA_10k_PEG_5k_ was equipped with MLN-4760 (MLN), a potent angiotensin-converting enzyme 2 (ACE2) inhibitor,^23^ that enables selective target cell recognition (Figure 2A, right) via binding to ACE2 thus fixing the nanoparticle to the cell surface.^24^ The newly achieved spatial proximity should ultimately lead to CPP exposure and induce uptake exclusively into target cells. In this way, we aim to provide a simple but effective strategy for the selective utilization of the unique properties of CPPs and thus establish a favorable, receptor-independent way of nanoparticle uptake into target cells, which is presumably associated with less side effects (Figure 2B).^24^ While possible target tissues of the developed nanoparticles could be strongly ACE2-expressing compartments, including renal tubules, gall bladder, cardiomyocytes, male reproductive cells, ductal cells, eye, and vasculature,^25^ the presented technology is a variable platform technology that can be adapted to various applications by changing the targeting ligand attached to longer polymer chains.

## 2 Materials and Methods

### 2.1 Materials

Millipore water was generated by a Milli-Q water purification system (Millipore, Schwalbach, Germany). Unless otherwise stated, all chemicals and reagents were obtained from Sigma-Aldrich (Taufkirchen, Germany) in analytical grade. Dulbecco’s phosphate-buffered saline (PBS) was purchased from Sigma-Aldrich (Taufkirchen, Germany). Poly(ethylene glycol) (PEG)-carboxylic acid with a molecular mass of 5000 g mol^–1^ (COOH-PEG5k–OH) was sourced from Jenkem Technology USA, Inc. (Allen, TX). Poly(ethylene glycol)-methyl ether with a molecular mass of 5000 g mol^–1^ (MeO-PEG5k–OH) as well as Resomer RG 502, poly(d,l-lactide-*co*-glycolide) (lactide:glycolide 50:50; ester- or acid-terminated; *M*_w_ 7000–17,000 Da) (PLGA) were purchased from Sigma-Aldrich (Taufkirchen, Germany). *N*,*N*′-Diisopropylcarbodiimide (DIC) and Oxyma were sourced from TCI (Eschborn, Germany). 1-[Bis(dimethylamino)methylene]-1H-1,2,3-triazolo[4,5-*b*]pyridinium 3-oxide hexafluorophosphate (HATU) and *N*,*N*′-diisopropylethylamine (DIPEA) were obtained from ABCR (Karlsruhe, Germany). MLN-4760 was obtained from MedChemExpress (Monmouth Junction, NJ). The fluorescent dyes were purchased from Lumiprobe (Hannover, Germany). Protected amino acids Fmoc-l-Arg(Pbf)–OH, Fmoc-l-Lys(Boc)–OH, Fmoc-l-Tyr(tBu)–OH, Fmoc-l-Gln(Trt)–OH, and Fmoc-Gly-OH were purchased from Carbolution Chemicals (St. Ingbert, Germany). The cellulose dialysis membranes had a molecular weight cutoff of 6–8 kDa and were obtained from Spectrum Laboratories, Inc. (Rancho Domingues, CA). The frits had a pore size of 35 μm and were sourced from Roland Vetter Laborbedarf (Ammerbuch, Germany). The centrifugal devices for nanoparticle concentration were purchased from Pall Life Sciences (Portsmouth, U.K.) and had a molecular weight cutoff of 30 kDa. The infrared lamp was obtained from Medisana (Neuss, Germany), with a thermostat from PEARL.GmbH (Buggingen, Germany). Syringes were purchased from Braun (Melsungen, Germany). NMR spectra were recorded on a Bruker Avance-400 NMR spectrometer (Bruker). High-resolution mass spectrometry (HRMS) was performed on a Q-TOF 6540 ultrahigh definition (UHD) LC/MS system (Agilent Technologies) using an electrospray ionization (ESI) source or on an AccuTOF GCX GC/MS system (Jeol) using an electron ionization (EI) source. Preparative HPLC was performed with a system from Thermo Fisher (Waltham) with a binary pump HPG-3200BX and the detector VWD-3400RS. HEK293 cells were sourced from the German Collection of Microorganisms and Cell Cultures GmbH, DSMZ (Göttingen, Germany). Transfected, stably ACE2-expressing HEK293T cells were a kind gift from Prof. Dr. Ralf Wagner (Institute of Clinical Microbiology and Hygiene, University Hospital Regensburg). Fetal bovine serum (FBS) for the preparation of cell culture medium was purchased from Biowest (Nuaillé, France). For all microscopic experiments, the cells were seeded in 8-well microscopy slides from Ibidi (Gräfelfing, Germany). Dako Faramount Mounting Medium was obtained from Agilent Technologies (Santa Clara). LysoTracker Deep Red (LTDR) was purchased from Invitrogen, Life Technologies GmbH (Darmstadt, Germany).

### 2.2 Synthesis of Cell-Penetrating Peptides (CPPs)

To identify suitable CPPs for the sequential nanoparticle targeting concept in terms of particle stability, uptake improvement, and cytotoxicity, various potential CPP candidates were synthesized and characterized: oligoarginines^27^ of different lengths (arginine-4 (R4), arginine-7 (R7), and arginine-10 (R10)), TAT47–57 (TAT) (one of the most characterized fragments of the human immunodeficiency virus (HIV) transactivator protein),^28^ and the Bax-inhibiting peptide (Bip) VSALK (small hydrophobic CPP, described by Gomez et al.^29^ (Table 1)).

**Table 1:** Peptide Sequences and Theoretical Netto Charges

peptide	sequence	charge
Bip	VSALK	+1
arginine-4 (R4)	RRRR	+4
arginine-7 (R7)	RRRRRRR	+7
TAT47–57 (TAT)	YGRKKRRQRRR	+8
arginine-10 (R10)	RRRRRRRRRR	+10

The corresponding results are provided in the Supporting Information (Figures S1–S20). The synthesis was performed using a standard Fmoc strategy following the procedure of Bresinsky et al.^30^ The 2-chlorotrityl-resin (300 mg, 1 equiv) was weighted into a fritted 25 mL syringe. 15 mL of dichloromethane (DCM) was drawn up to swell the resin at room temperature (RT) for 30 min. Afterward, DCM was aspirated with a vacuum flask. The first amino acid (2.5 equiv) was dissolved in the smallest possible volume of DCM (ca. 15 mL). If this was not possible, small amounts of dimethylformamide (DMF) were added until the amino acid was completely dissolved. 140 μL (2.5 equiv) of 2,4,6 collidine was added and the solution was drawn up into the syringe and shaken for 3 h at room temperature. After aspiration of the solvent, the resin with the bound amino acid was rinsed three times with 15 mL DCM. 15 mL of a mixture of piperidine 20% (V/V) in DMF was drawn up and the syringe was shaken on an orbital shaker at 35 °C for 15 min to remove the N-terminal Fmoc-protecting group. During this, the shaker was covered with a box, insulated with aluminum foil, while the temperature was adjusted with an infrared lamp. Temperature was controlled by a thermostat. After deprotection, the liquid was removed using a vacuum flask and the residual resin was washed three times with 15 mL of DMF. For the following coupling steps, the corresponding amino acid (2.5 equiv) and HATU (400 mg, 2.5 equiv) were weighed into two separate Erlenmeyer flasks. Afterward, both were dissolved in 5 mL of DMF and collidine (140 μL, 2.5 equiv) was added to the solution of HATU. Then, both solutions were drawn up with the resin-loaded syringe and shaken at 35 °C for 60 min. The liquid was again removed using a vacuum flask followed by three washing steps with DMF. For the synthesis of the Bax-inhibiting peptide (Bip) VSALK, different coupling reagents were used: HATU was replaced by Oxyma (2.5 equiv) and *N,N*′-diisopropylcarbodiimide (2.5 equiv) and no base additive was used for coupling. The coupling step was followed by the deprotection step with piperidine 20% (V/V) in DMF. Both reactions were repeated until the desired CPP sequence was built up. The last step was the cleavage from the resin. After deprotection of the last amino acid, the syringe was rinsed with methanol (2 mL × 15 mL), DCM (2 mL × 15 mL), and diethyl ether (2 mL × 15 mL) and allowed to dry. The resin was poured into a round-bottom flask and a solution of hexafluoroisopropanol (HFIP) in DCM (20%) was added dropwise. After stirring for 2 h, the solution was filtrated and the filtrate was evaporated and analyzed via NMR, HPLC, and mass spectrometry. If necessary, the reaction products were purified by preparative HPLC (Scheme S1).

### 2.3 Polymer Synthesis and Ligand Coupling

The synthesis of the PLA–PEG block copolymer was performed according to Qian et al.^31^ with modifications as previously described by our group.^32^ Directly prior to the polymerization reaction, 3,6-dimethyl-1,4-dioxane-2,5-dione was recrystallized from ethyl acetate at 85 °C and subsequently dried under vacuum at 38 °C overnight. The heterobifunctional PEG polymer COOH-PEG_2k_–OH, COOH-PEG_5k_–OH, or MeO-PEG_2k_–OH, respectively, served as macroinitiator for the reaction and were dissolved in 10 mL of anhydrous DCM in a round-bottom flask (1 eq, 0.19 mmol). 1,8-Diazabicyclo[5.4.0] undec-7-ene (DBU) (3 eq, 0.57 mmol) was added, the round-bottom flask was fitted with a drying tube, and the reaction mixture was stirred for exactly 1 h at RT. Subsequently, the polymerization reaction was quenched with benzoic acid (10 eq, 1.92 mmol). The reaction product was precipitated in 100 mL of ice-cold diethyl ether and dried under nitrogen flow overnight at RT. The resulting polymers were characterized via ^1^H NMR (Figures S21–S23). For the coupling of CPPs, PLA_10k_PEG_2k_COOH (4.165 μmol, 50 mg) was dissolved in anhydrous DMF. *N*-Hydroxysuccinimide (NHS) (25 equiv) and *N*-(3-(dimethylamino)propyl)-*N*′-ethylcarbodiimide hydrochloride (EDC) (25 equiv) were added as powder and the polymer was activated for 1 h at room temperature (RT) under stirring. The excess of EDC was quenched by the addition of β-mercaptoethanol (14.3 M) (BME) (35 equiv) for 15 min at RT. The protected CPP (3 equiv) was solved in 1000 μL of DMF and added to the stirring polymer solution. Simultaneously *N,N*′-diisopropylethylamine (DIPEA) (10 equiv) was added and the reaction was stirred for 24 h at RT. Afterward, the reaction product was precipitated into 100 mL of ice-cold diethyl ether and dried under nitrogen flow. For the peptide synthesis and to enable the specific coupling to the polymer via the N-terminus afterward, all amino acids had to be used side-chain-protected. Therefore, it was necessary to deprotect the peptide sequence after polymer modification. As the deprotection time in acidic environments should be kept as short as possible, due to hydrolysis instabilities of PLA–PEG polymers in acidic environments,^33,34^ the minimal reaction time for the cleavage of all protection groups was evaluated in advance (Supporting Information Chapter 4). According to these results, the modified polymers were dissolved in a mixture of 10 mL of dichloromethane (DCM) and 10 mL of trifluoroacetic acid (TFA) and stirred at room temperature for 60 min (Scheme S2). The solvent was removed on a rotary evaporator, and the polymer with the deprotected CPP was dried using a vacuum pump. The reaction product was dissolved in as little acetonitrile (ACN) as possible and added dropwise into a 10-fold excess of vigorously stirred Millipore water to generate polymeric micelles. The solution was stirred for 3 h under a fume hood to evaporate the organic solvent. Unreacted CPP and reagents were removed by dialysis of the polymeric micelles in a dialysis tube with a molecular weight cutoff of 6–8 kDa against 4 L of Millipore water with medium change after 30 min, 2 h, and 6 h. The coupling of MLN-4760 to PLA_10k_PEG_5k_COOH was performed according to Walter et al.^24^

### 2.4 Nanoparticle Preparation and Characterization

Ester-terminated 13.4 kDa PLGA as nanoparticle core component and (modified) PLA–PEG block copolymers constituting the nanoparticle shell were dissolved at a 30:70 mass ratio to a final concentration of 10 mg/mL in ACN. Various ratios of modified and unmodified shell polymers were investigated, which are precisely defined for the respective experiments and referred to as the degree of modification (DOM). The DOM corresponds to the mass fraction of PEG–PLA block copolymer with a CPP attached compared to the total shell polymer used for particle preparation; in other words, a DOM of 30% implies 30% CPP-modified PEG–PLA-block copolymer and 70% uncharged, methoxy-terminated PEG–PLA block copolymer. Nanoparticles were prepared via bulk nanoprecipitation. Therefore, the polymer mixtures were added dropwise into vigorously stirring 10% PBS to a final polymer concentration of 1 mg/mL. The preparation was stirred for 3 h at room temperature and if necessary concentrated by centrifugal ultrafiltration in filters with a molecular weight cutoff of 30 kDa for 20 min at 3000 *g*. For the preparation of fluorescently labeled nanoparticles, the core component PLGA was functionalized according to Walter et al.^24^ For the preparation of shielded particles, TAT47–57 was attached to PLA_10k_PEG_2k_ block copolymer and covered by PLA_10k_PEG_5k_ polymers modified with the potent and selective ACE2 inhibitor MLN-4760.^24^ Nanoparticles with methoxy-terminated long polymer chains and consequently without a specific surface modification, served as negative control. Both nanoparticle types were prepared with varying amounts of TAT to identify the most suitable CPP surface density. The amount of longer polymer chains was set to 25% since the particle design was based on a previous work of our group where nanoparticles with this surface density of MLN proved excellent particle avidities in the low nanomolar to picomolar range.^24^ ζ-Potential (ZP) and polydispersity index (PDI) were evaluated using a Malvern Zetasizer Nano ZS (Malvern, U.K.) by dynamic light scattering (DLS). The samples were analyzed with a 633 nm He–Ne laser at an angle of 137° at RT. Nanoparticle size and concentration were determined using nanoparticle tracking analysis (NTA) (NanoSight NS300, Malvern, U.K.). For NTA analysis, particles were diluted with Millipore water to a particle concentration of 20–100 particles per frame.

### 2.5 Cell Culture

HEK293 cells were cultivated in Dulbecco’s modified Eagle’s medium (DMEM) with 10% FBS. For the cultivation of HEK293T cells, which were stably expressing ACE2, the same medium was used with blasticidin supplementation in a concentration of 10 μg/mL. L929 cells were cultured in Eagle’s minimum essential medium (EMEM) with 10% FBS.

### 2.6 Cytotoxicity Assay

The cytotoxicity evaluation for CPP-modified nanoparticles was performed on L929 mouse fibroblasts because of their reproducible growth rates and biological responses.^35^ 10,000 cells/well were seeded in a 96-well plate and incubated for 24 h at 37 °C. The nanoparticle solutions were diluted in serum-containing medium and 100 μL per well were added. The cells were incubated for 24 h before the nanoparticle solution was aspirated. 200 μL of 3-(4,5-dimethylthiazol-2-yl)-2,5-diphenyltetrazolium bromide (MTT) solution was added and incubated for 6 h. MTT solution was prepared by weighing the solid and solving it in PBS to a concentration of 2.5 mg/mL. The solution was sterile-filtered and afterward diluted to a concentration of 0.625 mg/mL with medium containing FBS. After the incubation time, the supernatant was aspirated and 60 μL of a solution of 10% sodium dodecyl sulfate (SDS) in PBS was added. The 96-well plate was sealed with parafilm and stored overnight in the fridge. The next day the plate was shaken for 5 min on a plate shaker and absorption was measured at 570 and 690 nm at a Synergy Neo2Multi-Mode Microplate Reader (Biotek Instrument, Inc., Winooski, VT). For the evaluation, the difference in absorbance at 570 and 690 nm was analyzed and the results were normalized to untreated cells.

### 2.7 Flow Cytometry

HEK293 cells or HEK293T cells stably expressing ACE2 were seeded into 24-well plates at a concentration of 250,000 cells/well and incubated for 24 h at 37 °C. For coculture experiments, HEK293T-ACE2 cells were previously stained with Cell Tracker Green (CTG). Therefore, the cells were incubated for 45 min at 37 °C in a 15 μM solution of CTG in serum-free DMEM. Subsequently, 50,000 HEK293T-ACE2 cells and 50,000 HEK293 cells/well were seeded and incubated for 48 h at 37 °C. Both cell types were differentiated during measurements using the fluorescein (FITC) channel. NPs were prepared using Cy5-labeled PLGA and adjusted to 100 pM by diluting the particles with Leibovitz Medium (LM). The cell medium was aspirated and 300 μL of particle solution was added to each well. After 60 min incubation at 37 °C, the particle solutions were aspirated, and the cells were detached using 300 μL of a 0.04% trypsin/ 0.03% EDTA solution. All following work steps were carried out on ice. After all cells were detached, 700 μL of DMEM supplemented with 10% FBS was added to each well and the cells of each well were transferred to Eppendorf cups. These were centrifuged for 5 min at 200 *g* at 4 °C. The supernatant was aspirated and the cell pellets were washed with 1 mL of PBS. The centrifugation and aspiration step were repeated and the cell pellet was resuspended in 300 μL of PBS for flow cytometric measurement. The resuspended cells were stored on ice and protected from light until immediately before measurement. The samples were analyzed using a FACS Canto II (Becton Dickinson, Franklin Lakes, NJ). NP fluorescence was excited at 633 nm, and the emission was recorded using a 661/16 nm bandpass filter.

### 2.8 Confocal Scanning Microscopy (CLSM)

For confocal scanning microscopy (CLSM) analysis of the interaction of CPP-modified nanoparticles with cells, 50,000 cells per well were seeded in an 8-well ibidi slide and incubated for 24 h at 37 °C. The nanoparticle solutions were diluted to 100 pM in LM and 250 μL/well was added. The nanoparticles were incubated for 1 h at 37 °C. Afterward, the nanoparticle solution was aspirated and the cells were washed twice with 200 μL of prewarmed PBS. The cells were fixed directly after the second washing step or medium was added, respectively, and the cells were incubated overnight and fixed the following day. For cell fixation, a 4% paraformaldehyde (PFA) solution in PBS was used. PBS or cell medium was aspirated and 250 μL of PFA solution was added to each well. After incubation for 10 min at RT, the fixation solution was aspirated followed by two washing steps with PBS. Afterwards, the cells were incubated with DAPI staining solution (1 μg/mL in 0.1 M PBS) for 10 min for cell nucleus labeling. After two more washing steps with 200 μL of PBS, the cells were mounted using Dako Faramount Mounting Medium and stored in the fridge (4 °C) until measurement. For the investigation of nanoparticle uptake ways and endosomal escape, experiments with a LysoTracker Deep Red staining were additionally performed. Therefore, 250 μL of a nanoparticle solution of R7-modified NPs with a percentage of modified polymer of 50% in a concentration of 100 pM in DMEM + 10% FBS was added to the cells and incubated for 1 h at 37 °C. For this experiment, the nanoparticle core was labeled with 5-carboxytetramethyl rhodamine (TAMRA) as already described by our group.^24,32^ After the incubation period, the nanoparticles were aspirated and stained either directly or after a further incubation period of 24 h in fresh and particle-free medium. For staining, the cells were incubated with LysoTracker staining solution in a concentration of 50 nM for 1 h at 37 °C. Afterwards, the staining solution was aspirated and the cells were washed twice with 250 μL of fresh medium. Finally, 250 μL/well was added and the living cells were immediately analyzed by CLSM.

### 2.9 Data Analysis

#### 2.9.1 Statistics

Statistical analysis was performed using GraphPad Prism Software 6.0. Ordinary one-way ANOVA with a Dunnett’s (Figures 5 and 7) or Tukey’s (Figure 9 monoculture) multiple comparisons test and two-way ANOVA with a Sidak’s multiple comparisons test (Figure 9, coculture) was performed for statistical evaluation of significance. The number of performed experiments (*n*) and the resulting significance levels are indicated in the figure legends.

#### 2.9.2 Model for Data Fitting

The piecewise fitting model for ζ-potential data is presented in eq 1.
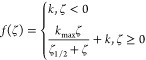
1

It yields an uptake constant for negative potentials *k* as well as a maximum uptake ratio *k*_max_ and a half-maximum uptake ζ-potential ζ_1/2_. The factors *k*_max_ and ζ_1/2_ describe (1) the potency of CPPs to increase cellular uptake and provide (2) an estimate of the threshold potential, above which further charges will not lead to a substantial improvement of uptake. Fitting of the piecewise model function was done using Origin (v. 10.1.0.178).

## 3 Results and Discussion

### 3.1 Systematic Evaluation of NPs Surface Modifications with CPPs

#### 3.1.1 Nanoparticle Preparation and Characterization

**Figure 3:**
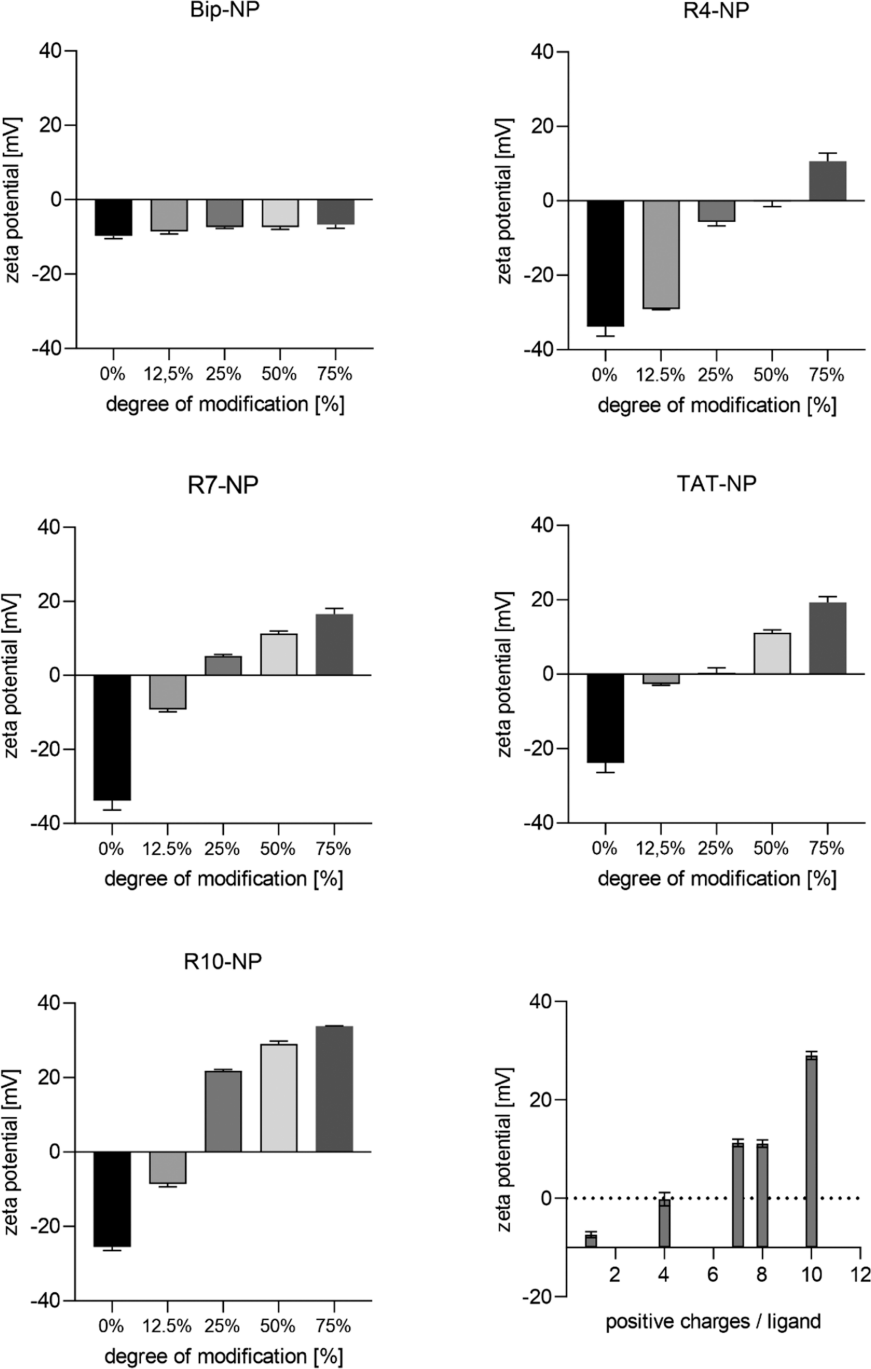
Characterization of the influence of nanoparticle modification with CPPs on their ζ-potential. The ζ-potential of nanoparticles modified with various CPPs (see diagram title) in different ratios of CPP-modified and unmodified methoxy-terminated polymer were analyzed. For all polycationic CPPs, increasing proportions of CPP-modified polymer led to increasing ζ-potentials. For the same DOM, a higher number of positive charges per ligand also led to higher absolute ζ-potentials (shown in the bottom right panel for a DOM of 50%) (*n* = 3 technical replicates).

The effect of various CPP candidates with different amounts of positive charges as well as the influence of CPP surface density on nanoparticle characteristics were investigated in a simplified nanoparticle design using only polymers of uniform lengths (PLA_10k_PEG_2k_). Control nanoparticles without CPPs attached to the NP surface had a size of 78 ± 1.8 nm. For CPP-modified particles, an increase in particle size was observed that varied between different CPPs and DOMs resulting in sizes between 90 and 130 nm. The PDI values of all particles were in the range of 0.1–0.2, which proved a narrow size distribution (Figure S28). Therefore, all modifications were shown to be suitable for an NP surface modification regarding particle stability. The nanoparticle ζ-potential served as a measure for the successful attachment of CPPs to particle surfaces (Figure 3). As expected, increasing the ratio of polycationic CPP-modified polymer to uncharged methoxy-terminated spacer polymer resulted in a corresponding shift of the ζ-potential to higher values. The net potential increase reached values of up to 59 mV, starting from a ζ-potential of approximately −25 mV at a DOM of 0% to +34 mV at a DOM of 75% for R10-modified particles. Bip, which is a hydrophobic cell-penetrating peptide with only one basic amino acid and thus only one positive charge,^36,37^ showed accordingly only a minor increase of 3 mV. Based on our results, a good correlation between the number of positive charges per ligand and the absolute value of the ζ-potential could be demonstrated for identical DOMFs.

#### 3.1.2 Qualitative Investigation of Nanoparticle Uptake

**Figure 4:**
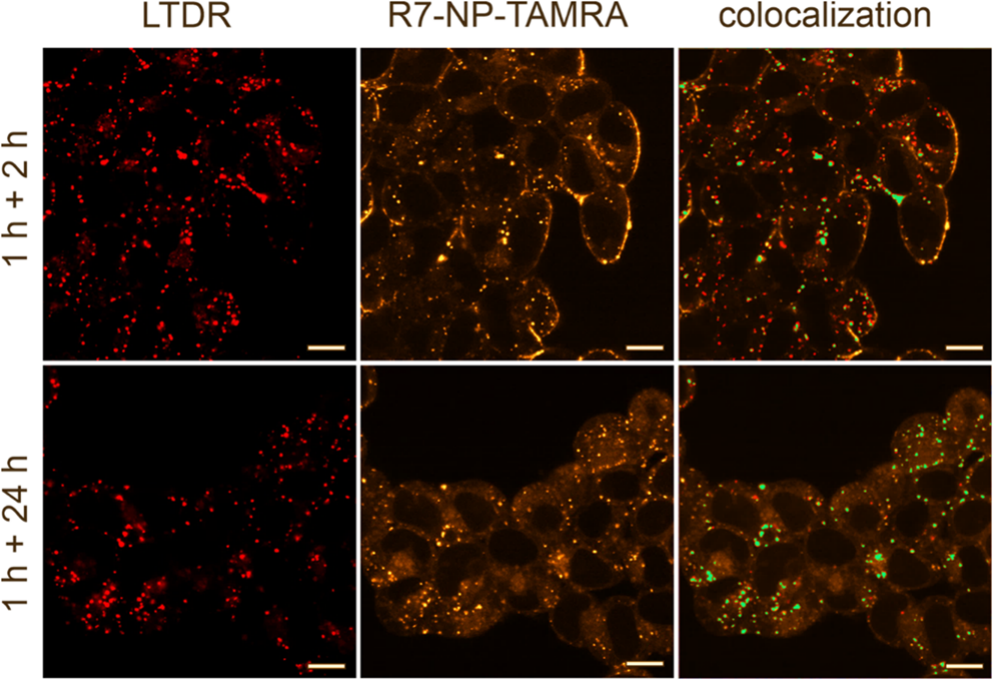
CLSM image of the qualitative evaluation of nanoparticle uptake. R7-modified nanoparticle binding and uptake were tracked after two different time points: the cells were incubated with NPs for 1 h. Afterward, the particles were removed, followed by further incubation for 2 h (1 h + 2 h) or 24 h (1 h + 24 h), respectively. Colocalization of LysoTracker deep red (LTDR), staining late endosomes and lysosomes (red), and R7-modified nanoparticles with a DOM of 50% labeled with TAMRA (R7-NP-TAMRA) (yellow) is shown in turquoise. The simultaneous employment of direct and endosomal uptake ways is presumed, since after 3 h, most particles still accumulate at the cell surface, but there is already background fluorescence visible inside the cell showing nanoparticle distribution in the cytoplasm. At this time point, almost no colocalization between particles and LTDR was visible, which indicates direct nanoparticle uptake. After 25 h, high endosomal entrapment was shown, which proves additional endosomal uptake. The increasing background fluorescence demonstrated that the CPP-modified particles reached the cytosol and therefore their destination inside the cell. Scale bar: 10 μm.

The qualitative investigation of particle uptake was performed for R7-modified particles as an example to prove their ability to reach the cytosol and qualify for intracellular drug delivery (Figure 4). For the advanced particle design with conditional CPP presentation and TAT as uptake signaling ligand, however, a corresponding internalization behavior was observed (Figure 9D). The R7-modified nanoparticles initially accumulated at the cell membrane (Figure 4). After 3 h, a faint fluorescence was visible in the cell interior, while there was almost no colocalization with endo- or lysosomes and most particles were localized outside the cell. After 1 day of incubation, the late endo- and lysosomes were occupied by nanoparticles to a high extent and the particles did no longer accumulate at the cell membrane. Additionally, the intracellular background fluorescence increased over time. These observations can be explained by uptake mechanisms of arginine-rich cell-penetrating peptides described in the literature: highly cationic structures strongly adsorb on membrane surfaces^38^ due to the interaction with membrane-associated proteoglycans including heparan sulfate (HSPG), which was reported to play a crucial role for the subsequent endocytic uptake via micropinocytosis.^38,39^ The observed fluorescence in the cytoplasm not colocalizing with endosomes indicates that a part of the nanoparticles was internalized via nonendocytic, direct uptake into the cytosol. This uptake mechanism was already postulated by different research groups for nanoparticles equipped with CPPs or high arginine surface densities.^40−42^ As clear evidence for such direct penetration served an energy-independent cell uptake at 4 °C, as well as the diffuse cytosolic labeling after treatment with micropinocytosis inhibitors,^43,44^ which showed that endocytosis-independent uptake pathways must be involved.^38,45^ However, the high colocalization of the nanoparticles and the lysotracker after 24 h (Figure 4) proved that endocytic uptake mechanisms played a major role in nanoparticle internalization. This is in line with the often described simultaneous prevalence of multiple internalization pathways.^38,46,47^ As a result, a certain number of NPs may suffer from endosomal entrapment and lysosomal degradation processes.^48−50^ Nevertheless, as the fluorescence in the cytosol increased considerably (Figure 4), it can be concluded that a decent share of nanoparticles can reach their destination and is, therefore, suitable for intracellular drug delivery.

#### 3.1.3 Quantitative Evaluation of Nanoparticle Uptake

**Figure 5:**
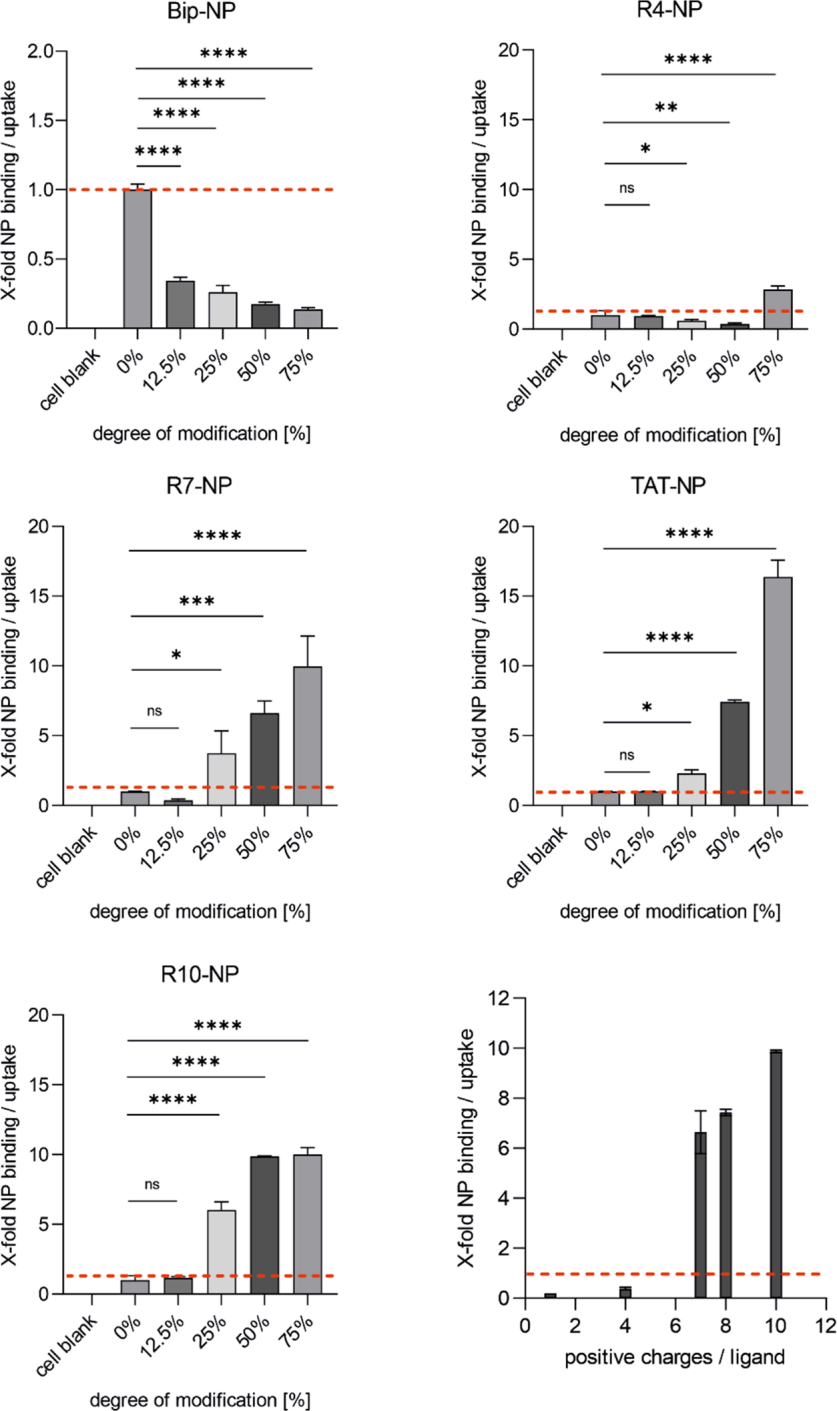
Comparison of nanoparticle binding/uptake improvement for different CPP modifications and different ratios of modified to unmodified polymer. The respective CPPs are indicated in the diagram headings. The panel at the bottom right shows the correlation between particle binding and uptake and the number of positive charges per ligand (DOM = 50%). Results represent mean ± standard deviation (SD) (*n* = 3, levels of statistical significance are indicated as *p ≤ 0.05, **p ≤ 0.01, ***p ≤ 0.001, ****p ≤ 0.0001).

The uptake-enhancing properties of the various CPP candidates were systematically analyzed (Figure 5). For R4-modified nanoparticles, the trend became apparent that higher surface densities were initially accompanied by a decrease in binding and uptake. This tendency reversed as soon as a certain minimum surface density was reached and the particles with a high DOM of 75% were taken up to a significantly higher amount than unmodified particles. Remarkably, this value correlated with the DOM, above which the R4-modified particles also showed a positive ζ-potential. This observation revealed the fact that a significant uptake improvement can be achieved with a relatively low number of positive charges per peptide, if the peptides are attached to the nanoparticle surface with a sufficiently high surface density, which compensates the missing peptide length. The CPPs with a higher number of positive charges per peptide, R7, TAT47–57, and R10, respectively, achieved uptake improvement even with a lower surface modification of at least 25% and increasing amounts of CPP surface density resulted in increasing nanoparticle binding and uptake, as expected. Increasing the amount of Bip was demonstrated to constantly decrease nanoparticle binding and uptake. In the literature, the uptake characteristics of small hydrophobic CPPs, which include various Bax-inhibiting peptides such as the Bip VSALK, are controversially discussed and poorly understood.^36,51^ There are different reports on their ability to transport cargos into the cell interior.^29,36,52^ However, since we did not observe any beneficial effects regarding nanoparticle uptake and it has already been shown that also scrambling of the peptide sequence does not significantly affect cellular uptake, we assume that other similar structures do not provide any advantages either.^19,52^ For this reason, this category of CPPs was not further considered for the establishment of nanoparticles with conditional CPP-mediated cell uptake.

**Figure 6:**
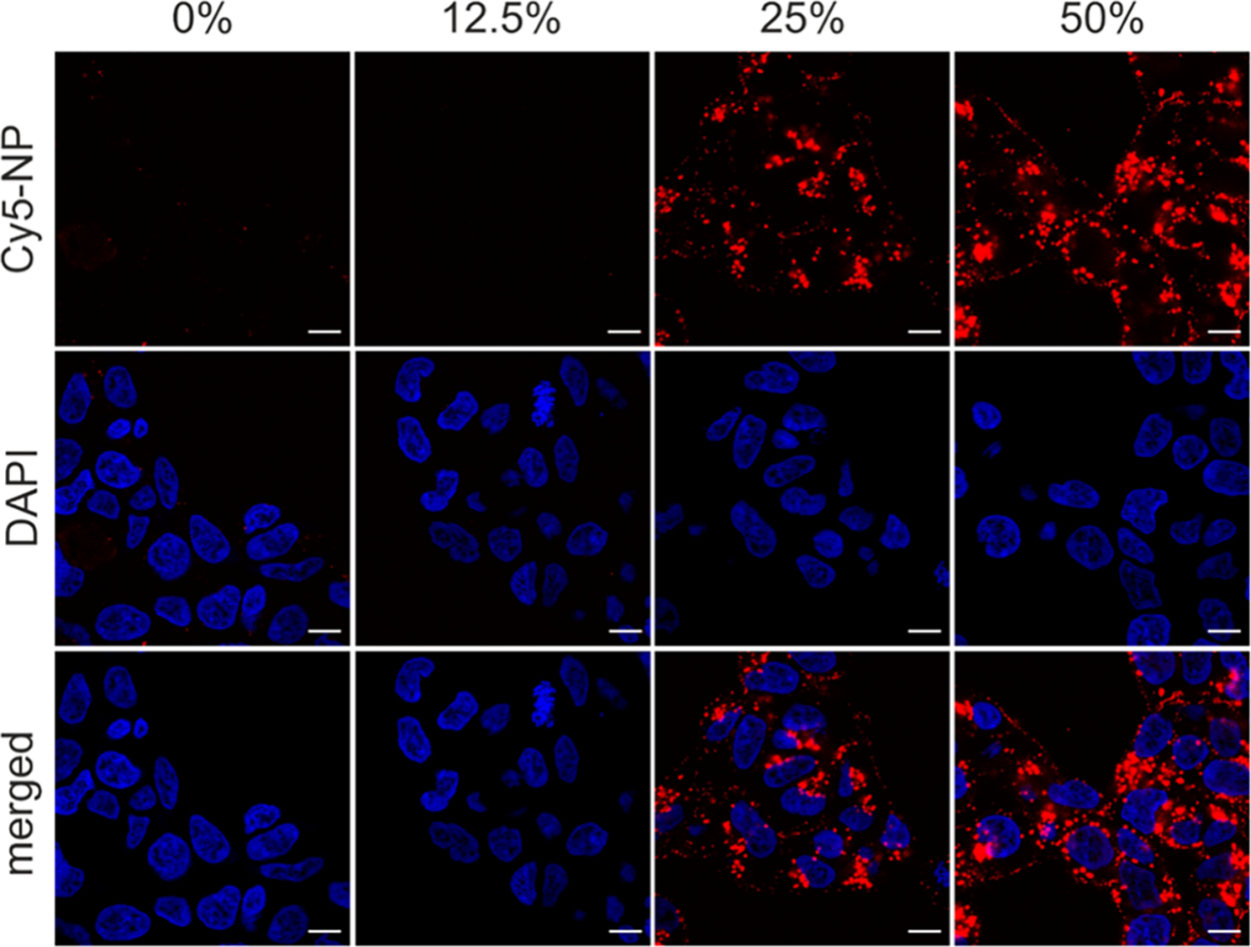
CLSM images of the uptake of Cy5-labeled R7-modified NPs with different DOMs in HEK293 cells. Cell nucleus labeling with DAPI (blue) served for cell localization. Increasing ratios of CPP-modified polymer mediated enhanced uptake of the Cy5-labeled nanoparticles (Cy5-NP) (red) into HEK293 cells. Scale bar: 10 μm.

The flow cytometric analysis findings were confirmed by CLSM for R7-modified particles as an example (Figure 6). The microscopic evaluation also verified the behavior of CPP-modified particles described in Section 3.1.2, i.e., that the surface modification with polycationic CPPs initially led to an increased accumulation at the cell membrane after 1 h of incubation. Therefore, the uptake properties were again investigated 16 h after particle incubation. At this time point, no more membrane accumulation was visible and most of the nanoparticles were taken up into the cells (Figure S29).

**Figure 7:**
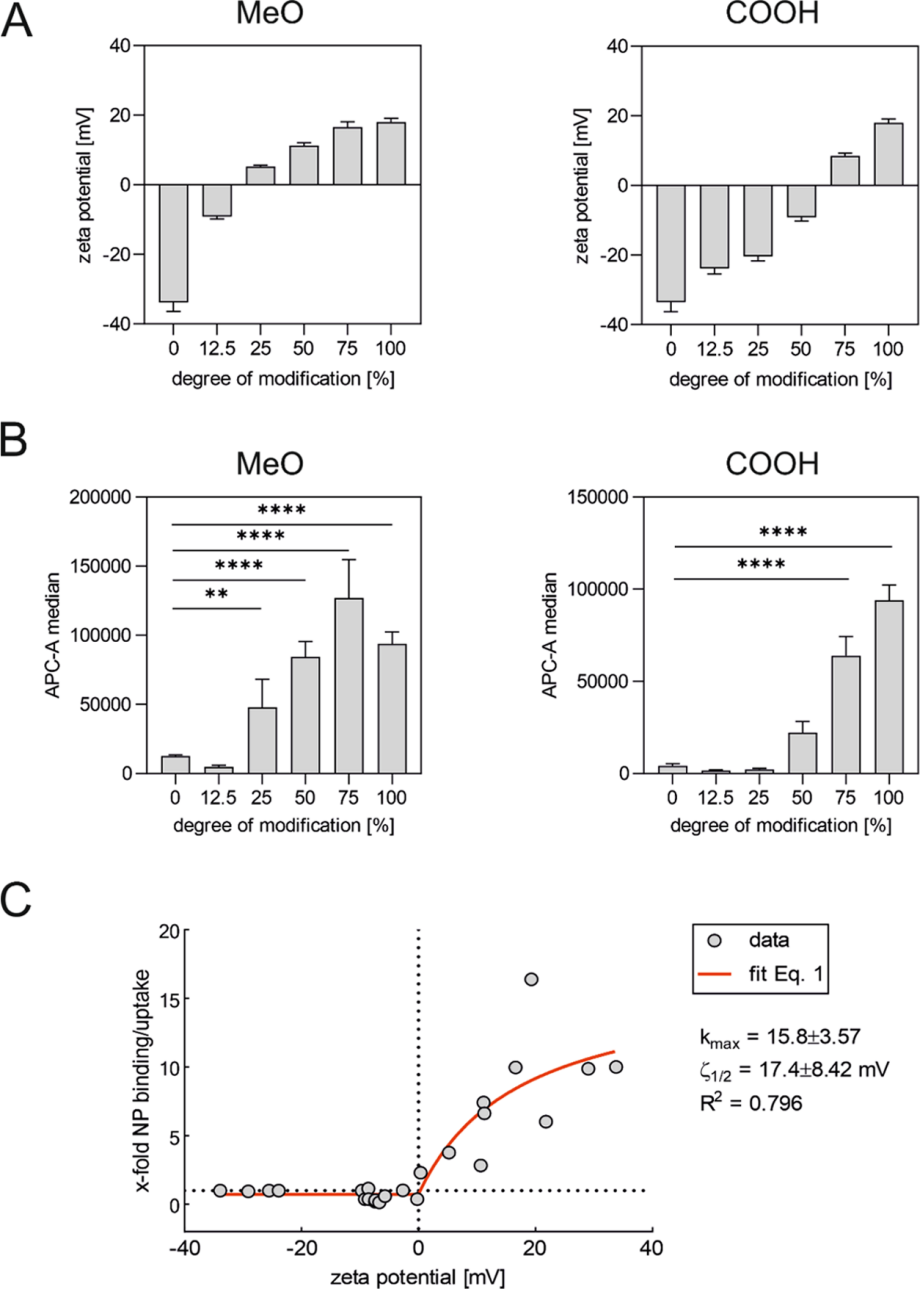
Correlation ζ-potential and uptake improvement of CPP-modified nanoparticles in HEK293 cells. The relationship between ζ-potential and uptake enhancement was verified using both an experimental (A, B) and a theoretical (C) approach. (A, B) NPs with identical CPP modifications (R7) and differently charged space-filling polymers (uncharged methoxy-terminated block copolymer (MeO) (left) and negatively charged carboxy-terminated block copolymer (COOH) (right)) were prepared. The APC-A median indicates the fluorescence signal induced by the binding or uptake of Cy5-labeled NPs in HEK293 cells. ζ-Potential measurements (A) and flow cytometric experiments (B) demonstrated that a positive ζ-potential was directly associated with a significant enhancement of particle uptake. (C) To quantitatively describe the relationship between surface charge and increased cellular uptake, a model was developed to deduct the maximum improvement of cellular uptake achievable (*k*_max_) by CPP functionalization and the ζ-potential required to obtain half-maximum uptake improvement ζ_1/2_. Results represent mean ± SD (*n* = 3, levels of statistical significance are indicated as *p ≤ 0.05, **p ≤ 0.01, ***p ≤ 0.001, ****p ≤ 0.0001); (AFU, arbitrary fluorescence units).

A higher theoretical net charge of the attached ligand was accompanied by a stronger influence on particle uptake, which was shown as an example of a constant DOM of 50% (Figure 5, bottom right). This is consistent with the literature, which describes an increase in cell penetration properties for longer arginine chains.^53^ However, this has not yet been quantitatively investigated for the modification of nanoparticle surfaces and the resulting changes in particle uptake. Additionally, this aspect suggests that there is not only a correlation between the theoretical net charge of the ligand but also between the total nanoparticle ζ-potential influenced by the CPP modification and its uptake. The preparation of nanoparticles with an equivalent surface density of R7 ligands, but negatively charged spacer polymers replacing the uncharged methoxy-terminated polymers, resulted in a shift to lower ζ-potentials. Therefore, higher CPP surface densities were necessary to reach a positive ζ-potential (Figure 7A). As expected, there was also a shift to higher DOMs for the significant uptake enhancement in HEK cells (Figure 7B). Only with 75% CPP modification, where a positive ζ-potential was detected, the particles showed a significantly increased uptake. Based on the ζ-potentials and corresponding uptake values of the different CPP modifications (Figures 3 and 5), we developed a model to quantitatively describe the relationship between surface charge and increased cellular uptake (Figure 7C). This allowed the assessment of a maximum improvement of cellular uptake of the described particle system (*k*_max_) achievable by CPP functionalization and the ζ-potential required to obtain half-maximum uptake improvement ζ_1/2_. We found that the CPP functionalization was capable of inducing an up to 15.8-fold increase of cellular uptake. Above a threshold potential of 17.4 mV, increasing charges will not lead to a substantial improvement of particle uptake. However, this correlation holds only as long as the CPP is directly visible on the particle surface. For more complex systems, where the CPP is shielded by longer polymers, it is no longer maintained (Figure S30). Nevertheless, the results are highly valuable for the development of CPP-modified nanoparticle design strategies since they allow for an initial assessment of whether a modification can ensure the desired cellular uptake by determining the nanoparticle ζ-potential.

Since high amounts of positive charges are also associated with cytotoxicity,^53,54^ a compromise must be found between uptake enhancement and cytotoxic effects. Our ζ_1/2_ value could serve as an orientation benchmark for this. R10-modified particles showed high cytotoxicity (Figure S31), which could be diminished, but not to a sufficient extent, via steric shielding (Figure S32). Therefore, TAT47–57 modification, which was associated with less cytotoxicity (Figure S33), was used for the following CPP-shielding experiments.

### 3.2 Conditional CPP-Mediated Nanoparticle Uptake in Target Cells

#### 3.2.1 Nanoparticle Characterization

**Figure 8:**
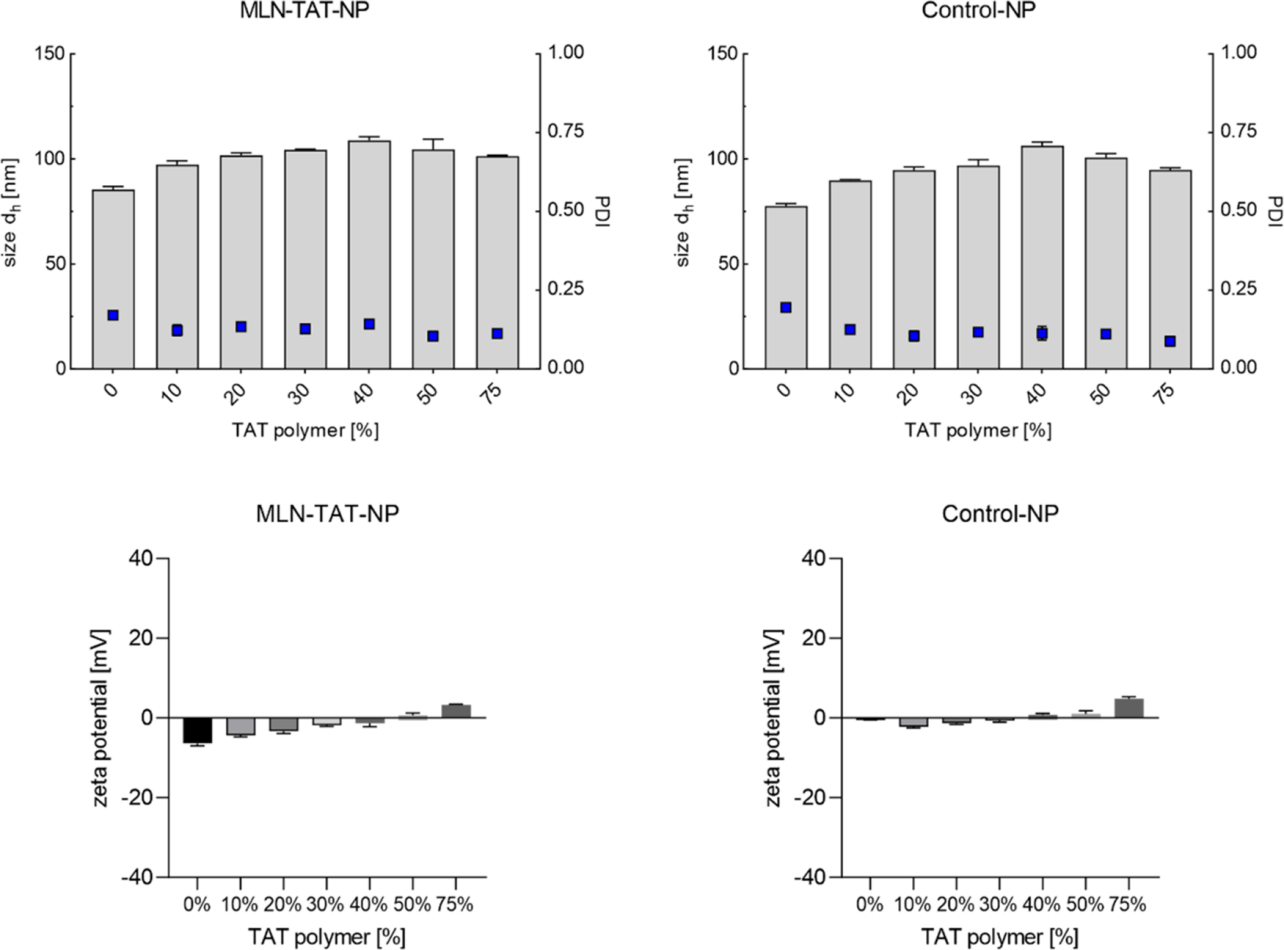
Characterization of nanoparticles with shielded CPP modification. The nanoparticles with TAT modification on short polymer chains (PEG2k) and MLN-4760 attached to long polymer chains (PEG5k) were characterized via DLS and NTA. Control NP contained methoxy-terminated longer polymers instead of the MLN-4760-modified polymers. The proportion of long polymers was set to 25%. The proportion of TAT-modified polymer varies and is indicated for every bar in the diagram (*n* = 3 technical replicates).

To enable a selective uptake improvement by CPPs attached to the nanoparticle surface, the polycationic uptake signal was sterically shielded by longer PEG chains. Control particles without TAT modification had a size of 77.6 ± 1.0 nm, which increased to values of 89.9 ± 0.3 to 106.4 ± 1.3 nm depending on the DOM. NPs with additional MLN modification were slightly larger and had a size of 85.4 ±1.3 nm without TAT modification and varying particle sizes of 97.4 ± 1.5 to 108.8 ±1.5 nm for different DOFs (Figure 8). However, due to the small differences in size, it can be assumed that there are no size-dependent differences in cell uptake. The nanoparticles with shielded CPPs showed similar PDI values to the unshielded particles between 0.1 and 0.2 and thus a narrow size distribution. Compared to the nanoparticles with CPPs directly visible on the NP surface, it was noticeable that the ζ-potential was hardly influenced by the positive charge of the polycationic ligands, since they were hidden inside the NP shell. Although the ζ-potential increased with rising TAT modification, positive ζ-potentials were only achieved at higher DOMs and the values were also significantly lower in absolute terms. For example, a DOM of 75% resulted in a ζ-potential of 4.84 ± 0.36 mV for the shielded particles, while without shielding polymers, the ζ-potential was markedly higher at 19.33 ± 1.21 mV. All MLN-modified particles had a slightly more negative ζ-potential than the unmodified control particles. This was caused by the negatively charged carboxy groups of MLN.

#### 3.2.2 Nanoparticle Binding and Uptake

**Figure 9:**
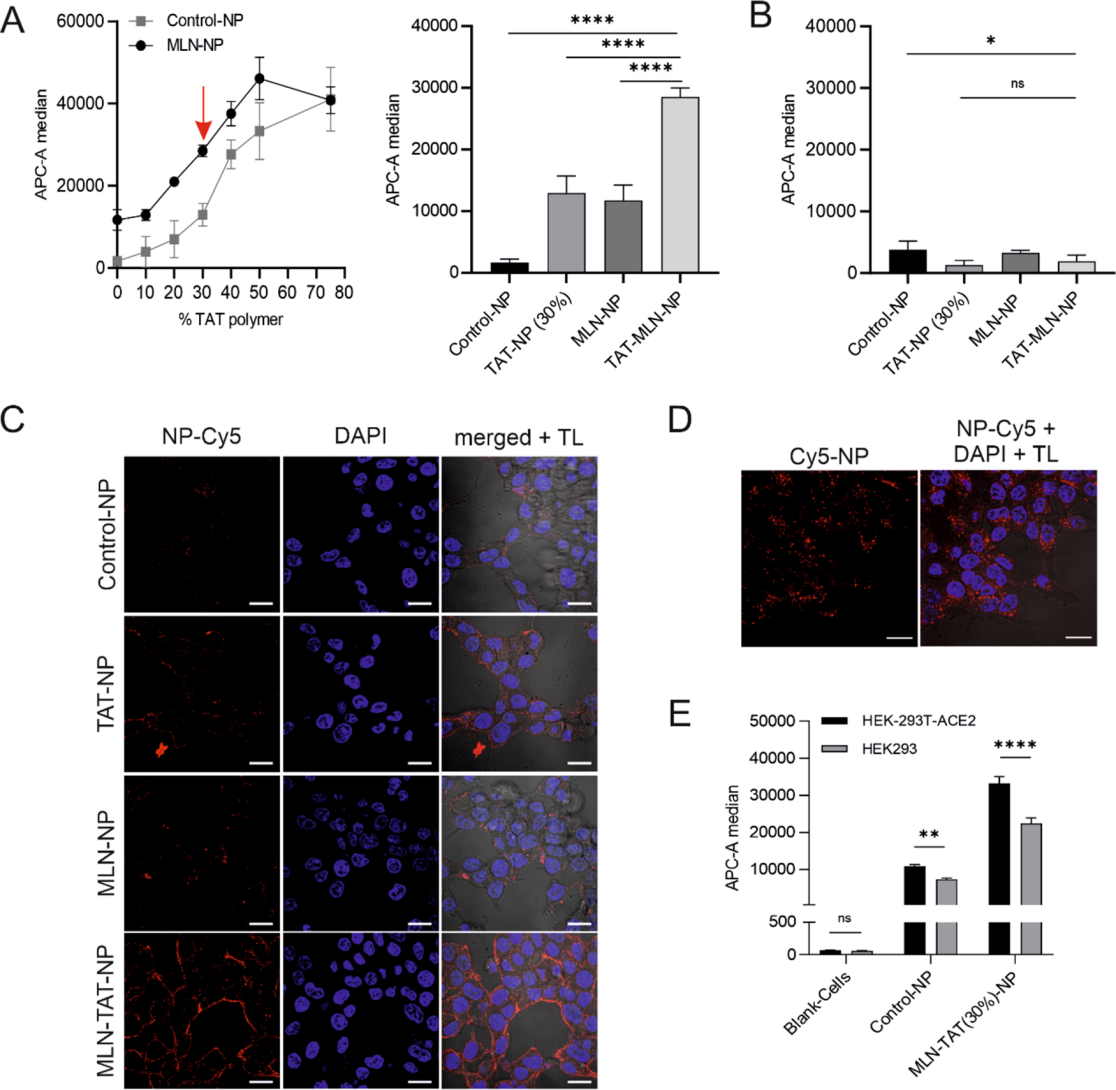
Flow cytometric and CLSM analysis of steric shielded NP binding and uptake. (A) Flow cytometric measurements of NP binding/uptake to ACE2-positive stable transfected HEK293T target cells. Different degrees of TAT47–57 functionalization (percentage of the shell polymer) were investigated and are stated on the *x*-axis to find a suitable CPP amount for the sequential targeting concept. The APC-A median shown on the *y*-axis represents the fluorescence of the cells corresponding to the binding/uptake of Cy5-labeled particles. A DOM of 30% was considered for the further experiments (red arrow). (B) Negative control with ACE2-negative HEK293 cells. (C) Confirmation of the results of (A) via CLSM analysis for a DOM of 30%. NPs were labeled with Cy5, cell nuclei were stained with DAPI. In the right row, the Cy5 and DAPI channels were merged and supplemented by transmitted light (TL) for cell localization. (D) CLSM evaluation of MLN-TAT-NP from (D) with further 24 h of incubation. (E) Coculture experiments with HEK293T-ACE2 stable cells and untransfected HEK293 cells. Scale bar: 20 μm. Results represent mean ± SD (*n* ≥ 3, levels of statistical significance are indicated as *p ≤ 0.05, **p ≤ 0.01, ***p ≤ 0.001, ****p ≤ 0.0001); (AFU, arbitrary fluorescence units).

Different amounts of TAT were examined to find a combination of the two ligands that ensures both high uptake and high selectivity. The MLN-modified particles bound reliably to a higher degree to the target cells than unmodified control particles up to a modification level of 50%. With a DOM of 75%, there was no significant difference between MLN-modified and control particles (Figure 9A). This was in line with our expectations, as the specific interaction itself led to increased binding, as demonstrated by the particles with MLN but without TAT modification (DOM 0%), and the spatial proximity increases TAT visibility. If the number of TAT ligands on the surface is too high, they led to a strong effect even without additional MLN surface modification. Therefore, a compromise must be found between the highest possible selectivity and the highest possible uptake in target cells. Based on our described preliminary experiments (Figure 9A), a combination of 25% MLN-modified polymer and a DOM of TAT of 30% was considered for further investigations. To ensure that the effect of enhanced binding with MLN attached to the NP surface results from specific binding to ACE2, the particle binding was additionally evaluated with untransfected HEK cells as off-target cell. In this case, neither the modification with MLN nor the dual modification led to improved particle uptake, which confirmed the target cell selectivity of the established concept (Figure 9B). The results found in flow cytometry were additionally investigated by CLSM measurements (Figure 9C). The microscopic evaluation supported the results and showed that although the modification with solely TAT or MLN also led to an improvement in particle binding to the target cells, this effect could be significantly increased by combining the ligands in the sequential targeting system. The fact that NP binding ultimately results in particle uptake into target cells was explicitly demonstrated for this case by further incubation of the cells after particle re-movement for 24 h (Figure 9D). To investigate target cell selectivity even in the presence of off-target cells, coculture experiments were performed. It could be demonstrated that the dual-modified particles are considerably superior in their ability to distinguish between target and off-target cells compared to control NP and thus bound to a higher extent to the surface of target cells (Figure 9E).

## 4 Conclusions

In this study, we systematically evaluated the effect of nanoparticle surface modifications with various cell-penetrating peptides. We identified correlations between the number of positive charges per peptide, surface density, ζ-potential, and the corresponding uptake enhancement in cell experiments, which is highly valuable for the implementation of nanoparticle design strategies containing cell-penetrating peptides. Based on these data, we established a targeting strategy, that allows us to utilize the unique uptake-enhancing properties of cell-penetrating peptides in a selective way by promoting a conditional cell internalization only after a prior selective cell binding has revealed the uptake signal. In conclusion, we have achieved a selective, completely receptor-independent nanoparticle uptake into target cells, which is expected to be beneficial with respect to targeting-related side effects and enables an exclusively cargo-dependent pharmacological effect.

## Supporting Information Available

Detailed synthesis scheme of side-chain-protected CPPs, ^1^H NMR, HPLC, and MS spectra for all compounds, characterization of PLA–PEG block copolymers, polymer modification, further NP characterization, detailed analysis of NP uptake behavior via CLSM, influence of CPP shielding on ζ-potential, and cytotoxicity evaluations of NPs (PDF)

## Abbreviations

ACE2: angiotensin-converting enzyme 2
ACN: acetonitrile
BME: β-mercaptoethanol
Bip: Bax-inhibiting peptide (VSALK)
CPPs: cell-penetrating peptides
CLSM: confocal scanning microscopy
CTG: cell tracker green
DBU: 1,8-diazabicyclo[5.4.0]undec-7-ene
DCM: dichloromethane
DIC: *N,N*′-diisopropylcarbodiimide
DIPEA: *N,N*′-diisopropylethylamine
DLS: dynamic light scattering
DMEM: Dulbecco’s modified Eagle’s medium
DMF: dimethylformamide
DOM: degree of modification
EDC: *N*-(3-(dimethylamino)propyl)-*N*′-ethylcarbodiimide hydrochloride
EMEM: Eagle’s minimum essential medium
EI: electoionization
ESI: electosprayionization
FBS: fetal bovine serum
FITC: fluorescein
HATU: 1-[bis(dimethylamino)methylene]-1H-1,2,3-triazolo[4,5-*b*]pyridinium 3-oxide hexafluorophosphate
HFIP: hexafluoro-2-propanol
HRMS: high-resolution mass spectrometry
HSPG: heparan sulfate
LM: Leibovitz medium
LTDR: LysoTracker deep red
MeO: methoxy
MLN: MLN-4760
MTT: 3-(4,5-dimethylthiazol-2-yl)-2,5-diphenyltetrazolium bromide
NHS: *N*-hydroxysuccinimide
NPs: nanoparticles
NTA: nanoparticle tracking analysis
PBS: Dulbecco’s phosphate-buffered saline
PEG: poly(ethylene glycol)
PFA: paraformaldehyde
PLA: poly(lactic acid)
PLA–PEG: block copolymer of poly(lactid acid)-poly(ethylene glycol)
PLGA: poly(d,l-lactide-*co*-glycolide)
R4: arginine-4
R7: arginine-7
R10: arginine-10
RT: room temperature
SD: standard deviation
SDS: sodium dodecyl sulfate
TAT: transactivator of transcription (47–57)
TAMRA: 5-carboxytetramethyl rhodamine
TFA: trifluoroacetic acid
TL: transmitted light
UHD: ultrahigh definition
ZP: ζ-potential
