# Supplementary material for: Conditional Cell-Penetrating Peptide Exposure as Selective Nanoparticle Uptake Signal
Source: ACS Appl Mater Interfaces. 2024 Jul 16;16(29):37734–47. doi: 10.1021/acsami.4c07821 (PMC11284753; doi:10.1021/acsami.4c07821)
Supplement: Supplementary file 1 — am4c07821_si_001.pdf [file am4c07821_si_001.pdf]

Supporting Information for

Conditional Cell Penetrating Peptide Exposure as Selective Nanoparticle Uptake Signal

Melanie Walter[a], Merlin Bresinsky[b], Oliver Zimmer[a], Steffen Pockes[b], Achim Goepferich[a]*

[a]Department of Pharmaceutical Technology, University of Regensburg, 93053 Regensburg, 93053, Germany.

[b]Department of Medicinal Chemistry I, University of Regensburg, 93053 Regensburg, Bavaria, Germany.

*Corresponding author
Email: achim.goepferich@ur.de
Phone: +49 941 943-4842

1. Synthesis of Side Chain-Protected Cell Penetrating Peptides

1.1 Detailed Synthesis Scheme of Side Chain Protected CPPs

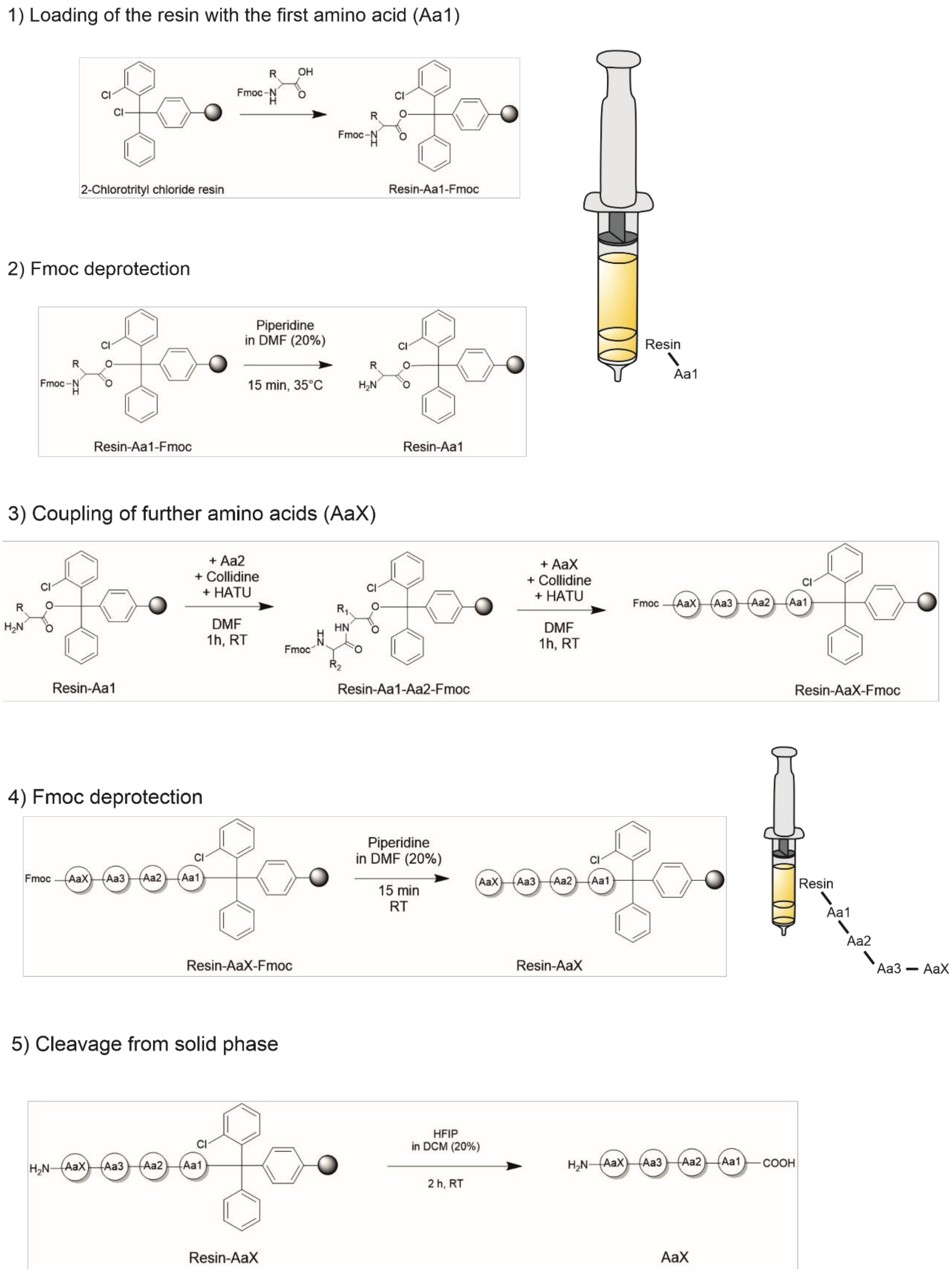

Scheme S1. Detailed synthesis scheme.

2 Analytics of Cell Penetrating Peptides

2.1 Bip$_{prot.}$

2.1.1 Structure

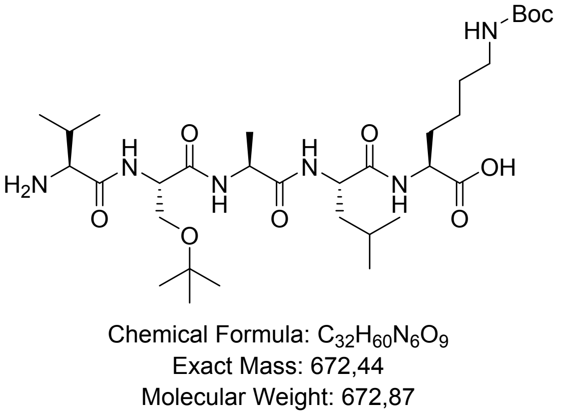

Figure S1. Structure of Bip$_{prot.}$.

2.1.2 NMR

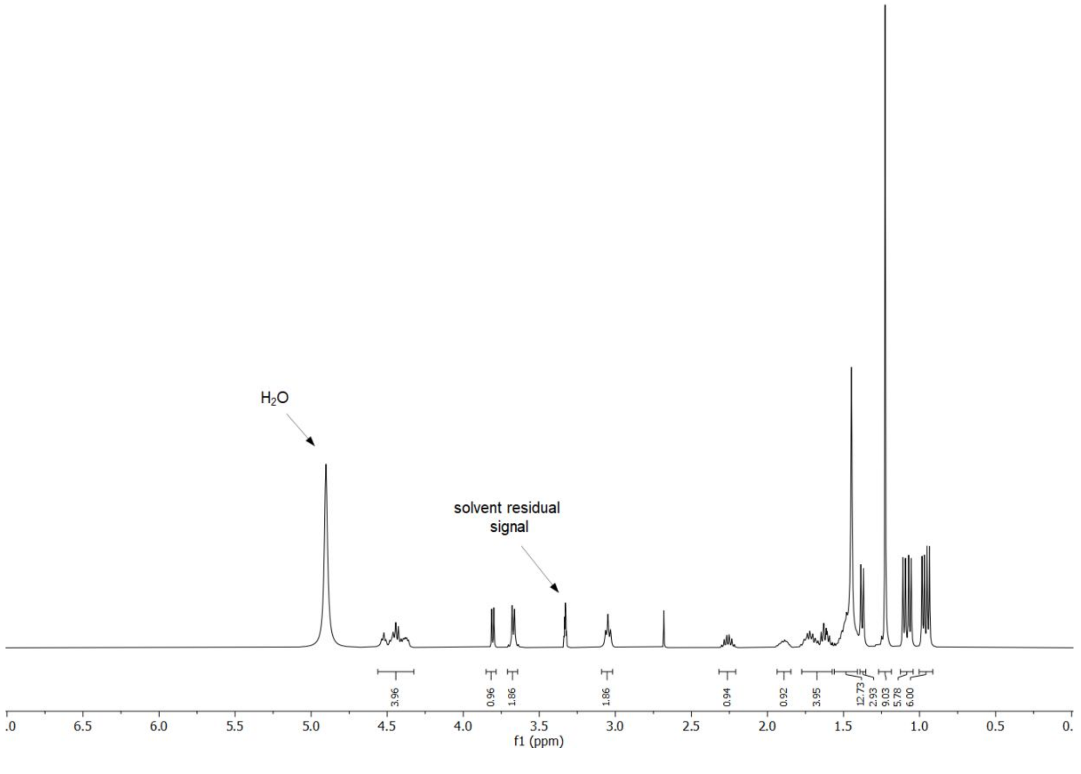

Figure S2. ^{1}H-NMR of Bip$_{prot.}$. ^{1}H-NMR (400 MHz, CD_3OD) δ 4.58 – 4.31 (m, 4H), 3.81 (d, *J* = 5.7 Hz, 1H), 3.67 (d, *J* = 5.9, 1.3 Hz, 2H), 3.05 (t, *J* = 6.7 Hz, 2H), 2.32 – 2.20 (m, 1H), 1.94 – 1.82 (m, 1H), 1.79 – 1.57 (m, 4H), 1.56 – 1.40 (m, 13H), 1.38 (d, *J* = 7.0 Hz, 3H), 1.23 (s, 9H), 1.08 (dd, *J* = 15.2, 6.9 Hz, 6H), 0.96 (dd, *J* = 13.3, 6.4 Hz, 6H).

2.1.3 *Mass spectrometry*

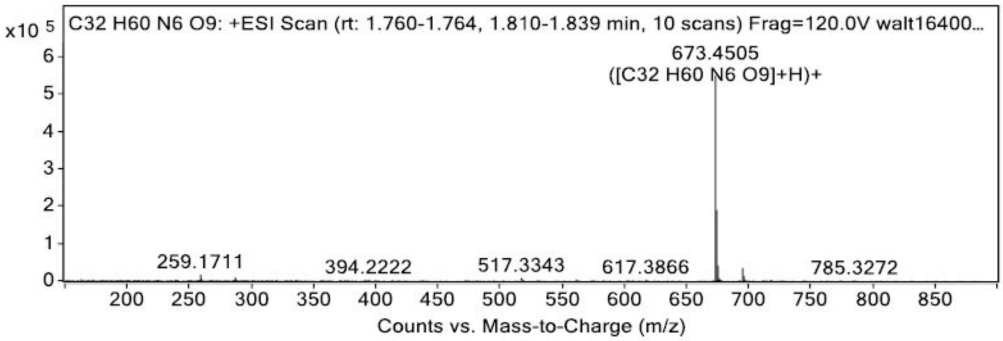

Figure S3. Mass spectrum of Bip$_{prot.}$. HRMS: (M+H) *m/z* calculated for $C_{32}H_{61}N_6O_9^+$: 673.4495, found: 673.4505.

2.1.4 *HPLC*

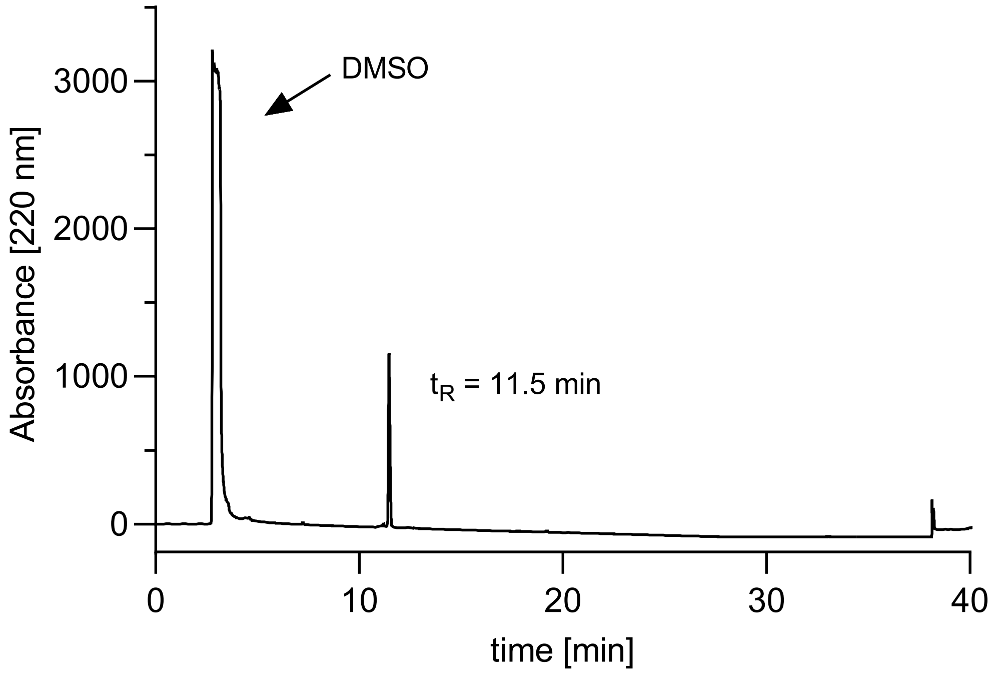

Figure S4. RP-HPLC analysis of Bip$_{prot.}$. Retention time t_R of Bip$_{prot.}$: 11.5 min.

2.2 R4$_{prot.}$

2.2.1 Structure

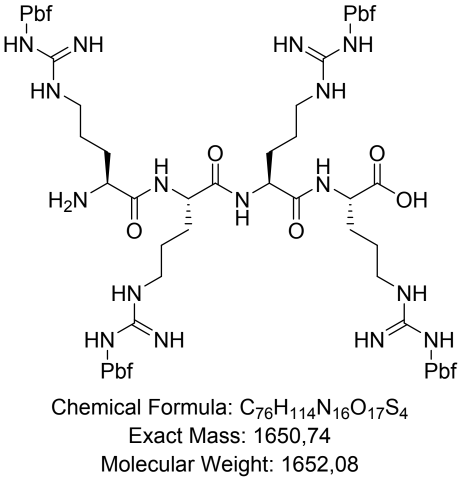

Figure S5. Structure of R4$_{prot.}$.

2.2.2 NMR

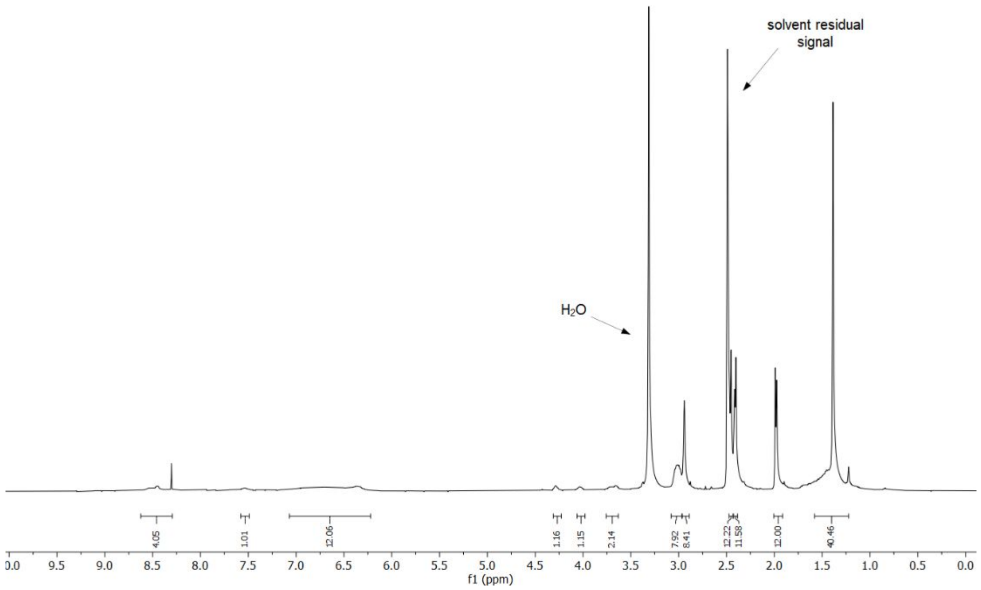

Figure S6. ^{1}H-NMR spectrum of R4$_{prot.}$. ^{1}H-NMR (400 MHz, DMSO-d6) δ 8.61 – 8.29 (m, 4H), 7.54 (s, 1H), 7.07 – 6.20 (m, 12H), 4.28 (s, 1H), 4.03 (s, 1H), 3.69 (d, J = 26.2 Hz, 2H), 3.08 – 2.96 (m, 8H), 2.94 (s, 8H), 2.47 – 2.43 (m, 12H), 2.42 – 2.39 (m, 12H), 1.98 (d, J = 6.2 Hz, 12H), 1.60 – 1.20 (m, 40H).

2.2.3 Mass spectrometry

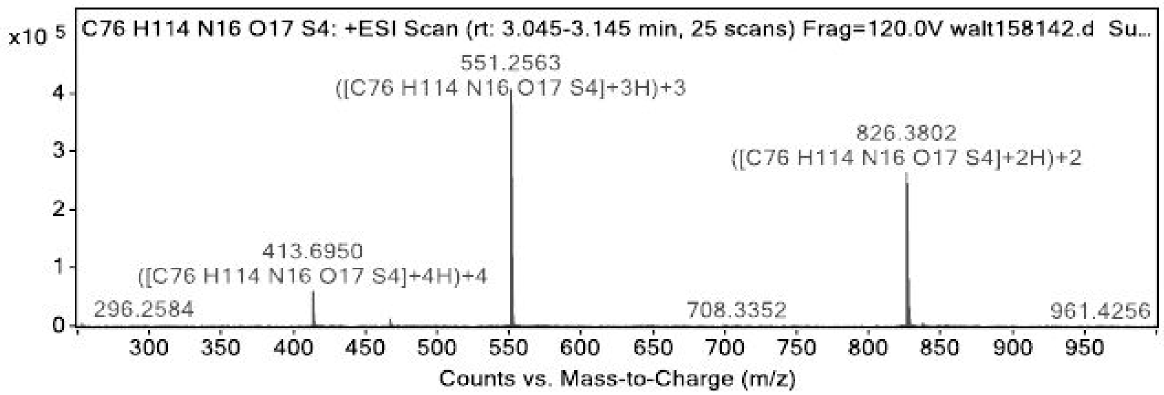

Figure S7. Mass spectrum of R4$_{prot.}$. HRMS: $(M+2H)^{+2}$ *m/z* calculated for $C_{76}H_{116}N_{16}O_{17}S_4^{2+}$: 826.3788, found: 826.3802.

2.2.4 HPLC

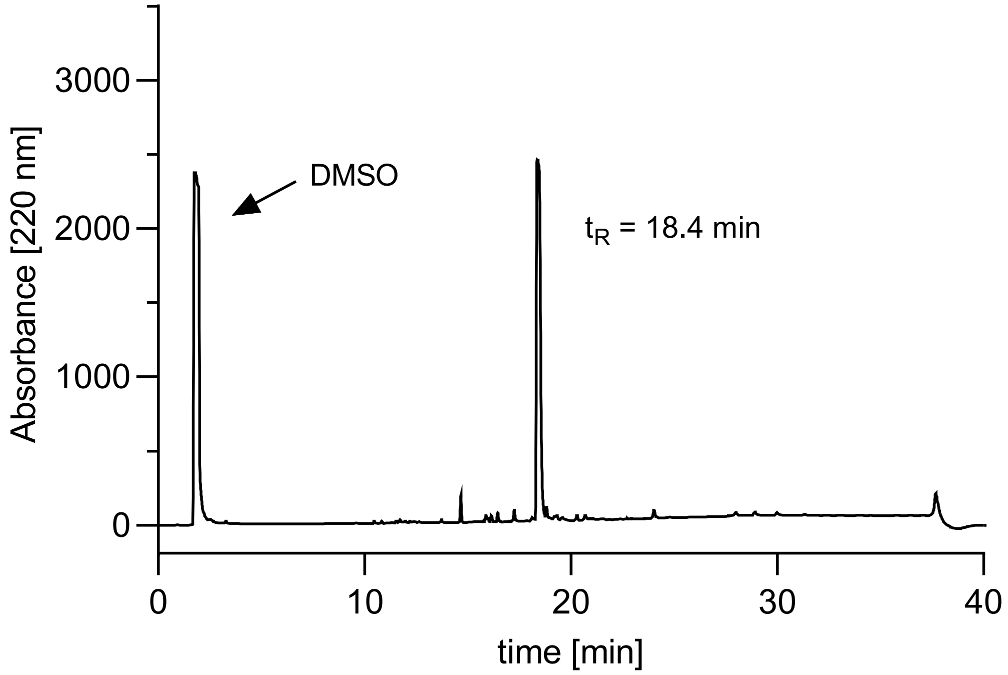

Figure S8. RP-HPLC analysis of R4$_{prot.}$. Retention time t_R of R4$_{prot.}$: 18.4 min.

2.3 R7$_{prot.}$

2.3.1 Structure

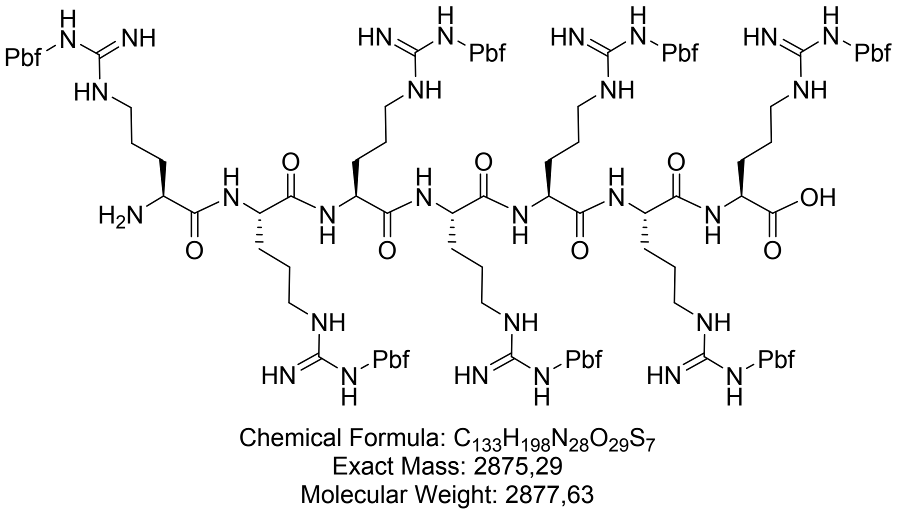

Figure S9. Structure of R7$_{prot.}$.

2.3.2 NMR

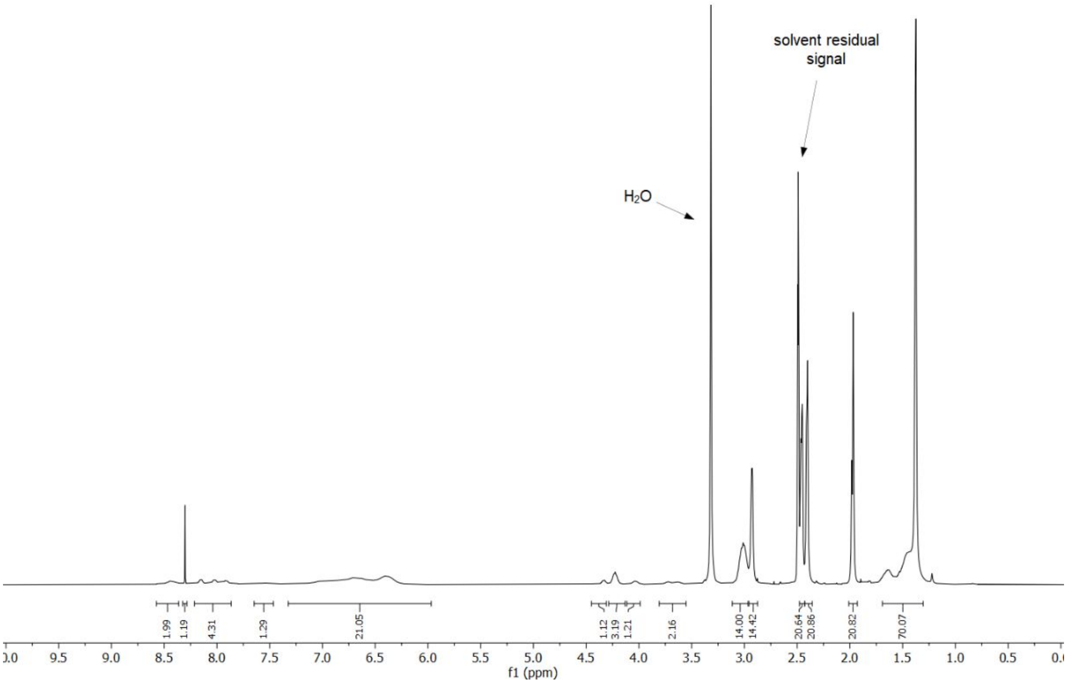

Figure S 10. ^{1}H-NMR spectrum of R7$_{prot.}$. ^{1}H-NMR (400 MHz, DMSO-d6) δ 8.43 (s, 2H), 8.30 (s, 1H), 8.21 – 7.83 (m, 4H), 7.50 (s, 1H), 7.31 – 6.06 (m, 21H), 4.44 – 4.30 (m, 1H), 4.27 – 4.14 (m, 3H), 4.03 (s, 1H), 3.67 (d, J = 37.5 Hz, 2H), 3.10 – 2.96 (m, 14H), 2.93 (s, 14H), 2.48 – 2.43 (m, 21H), 2.42 – 2.36 (m, 21H), 2.01 – 1.93 (m, 21H), 1.71 – 1.25 (m, 70H).

2.3.3 *Mass spectrometry*

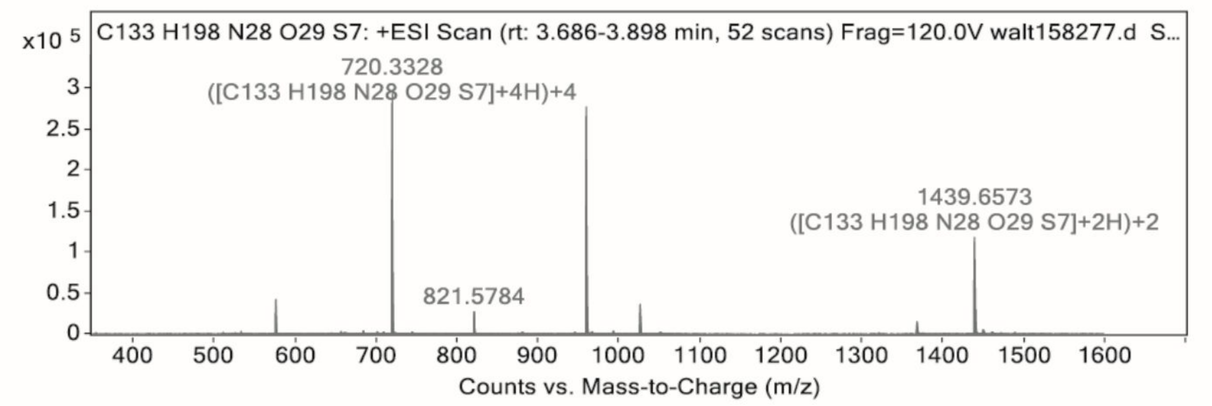

Figure S11. Mass spectrum of R7$_{prot.}$. HRMS: $(M+2H)^{+2}$ *m/z* calculated for $C_{133}H_{200}N_{28}O_{29}S_7^{2+}$: 1439.6554, found: 1439.6573.

2.3.4 *HPLC*

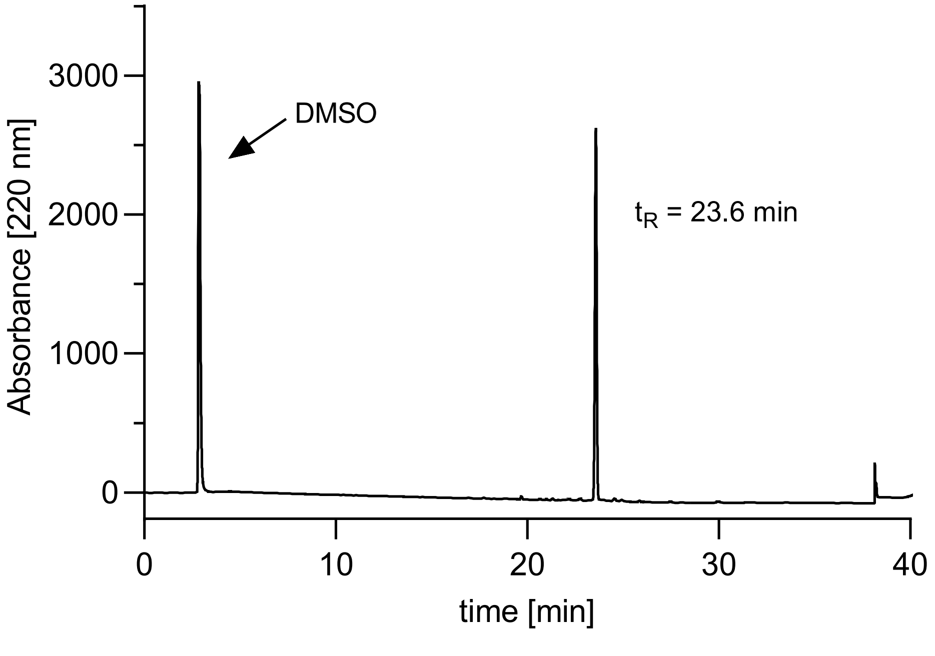

Figure S12. RP-HPLC analysis of R7$_{prot.}$. Retention time t_R of R7$_{prot.}$: 23.6 min.

2.4 TAT(47-57)prot.

2.4.1 Structure

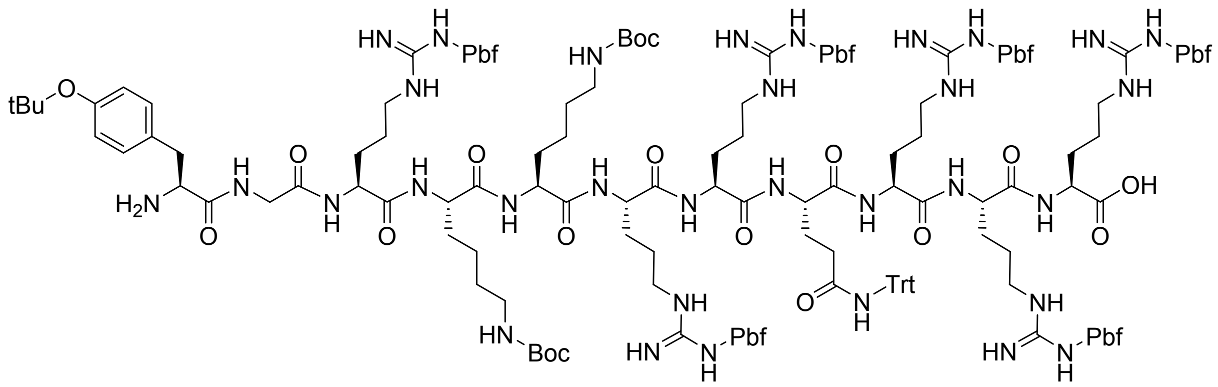

Figure S13. Structure of TAT(47-57)$_{prot.}$.

2.4.2 NMR

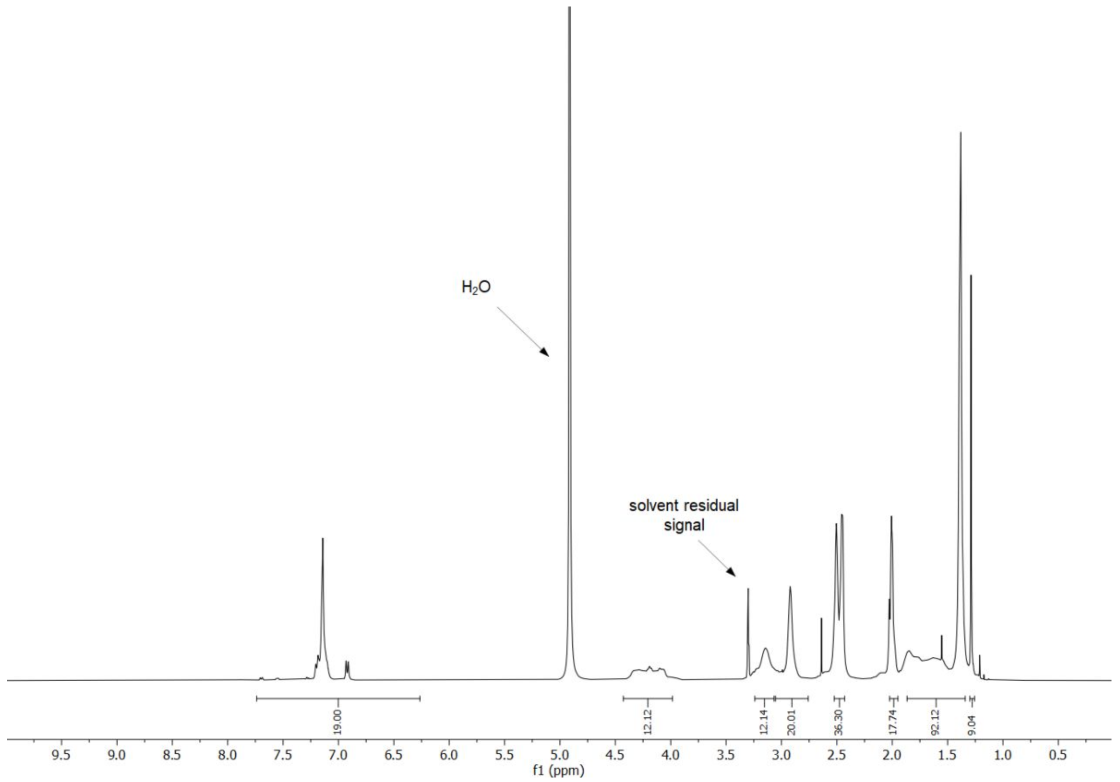

Figure S14. ^{1}H-NMR spectrum of TAT(47-57)$_{prot.}$. ^{1}H-NMR (400 MHz, CD_3OD) δ 7.72 – 6.26 (m, 19H), 4.41 – 3.92 (m, 12H), 3.23 – 3.07 (m, 12H), 3.03 – 2.80 (m, 20H), 2.52 – 2.41 (m, 36H), 2.05 – 1.94 (m, 18H), 1.88 – 1.34 (m, 92H), 1.29 (s, 9H).

2.4.3 *Mass spectrometry*

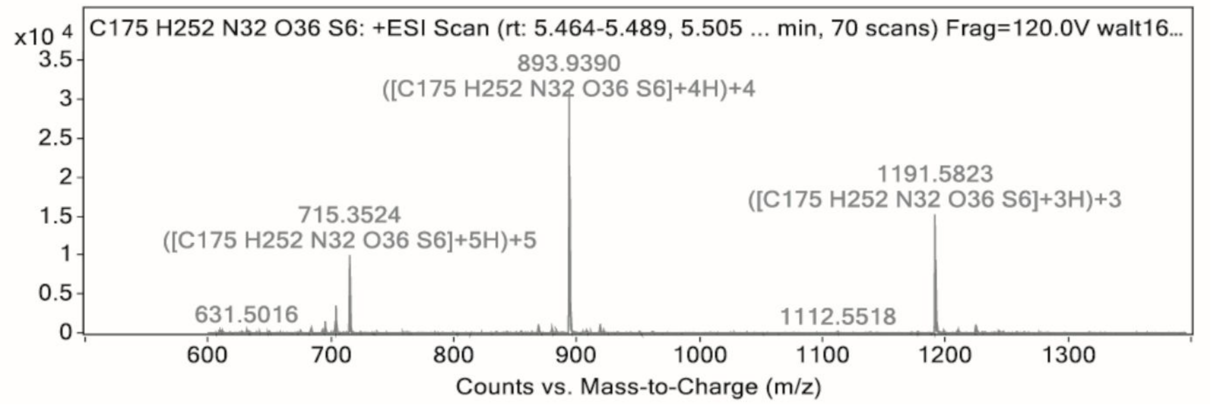

Figure S15. Mass spectrum of TAT(47-57)$_{prot.}$. HRMS: $(M+3H)^{+3}$ *m/z* calculated for $C_{175}H_{255}N_{32}O_{36}S_6^{3+}$: 1181.5823, found: 1191.5821.

2.4.4 *HPLC*

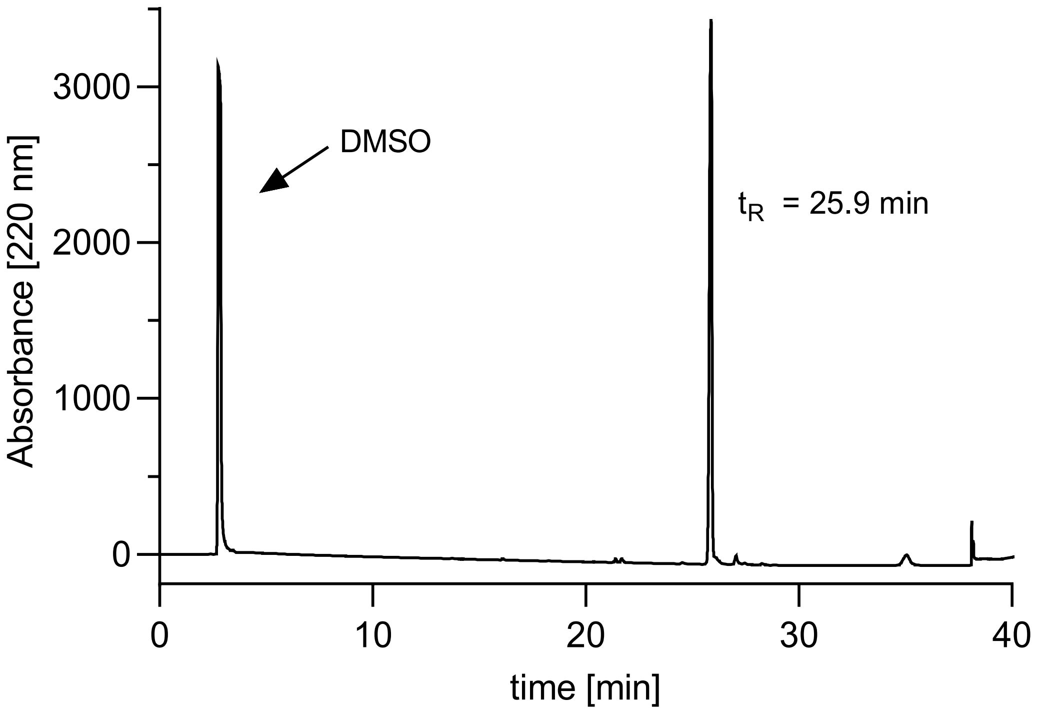

Figure S16. RP-HPLC analysis of TAT(47-57)$_{prot}$. Retention time t_R of TAT(47-57)$_{prot.}$: 25.9 min.

2.5 R10prot.

2.5.1 Structure

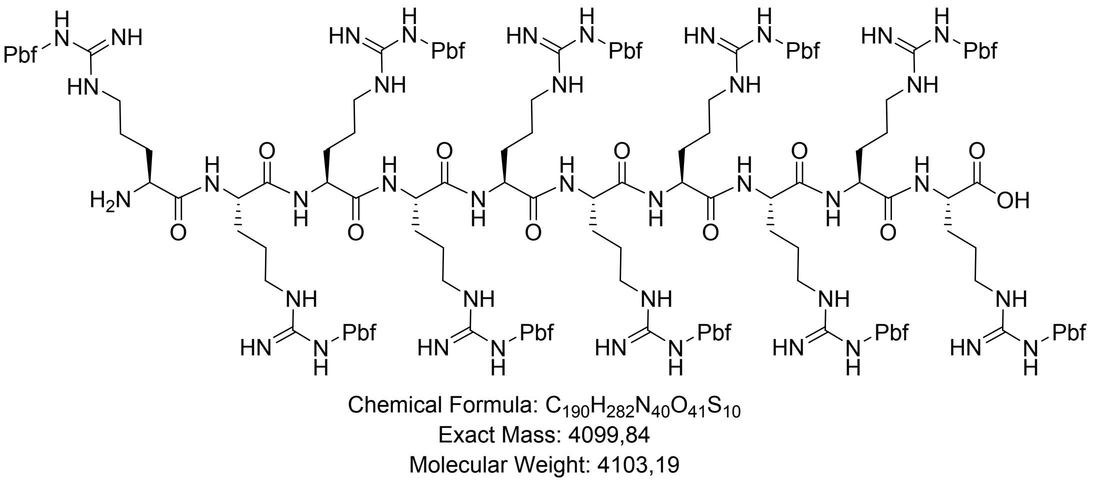

Figure S17. Structure of R10$_{prot.}$.

2.5.2 NMR

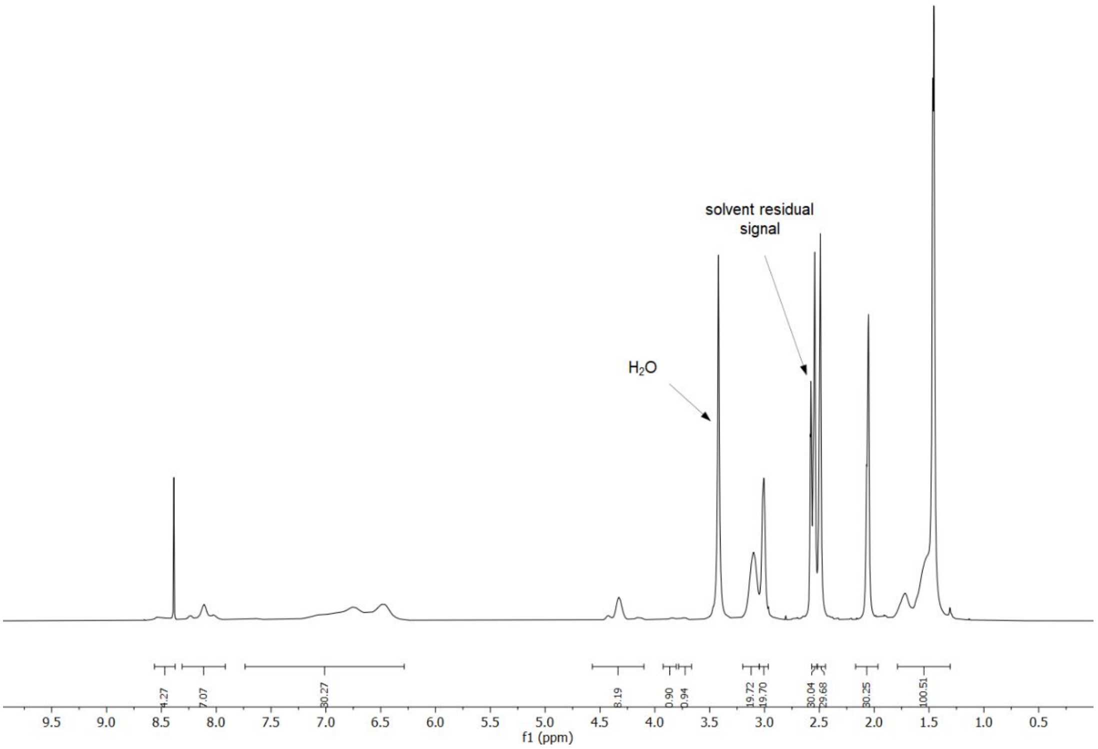

Figure S18. ^{1}H-NMR spectrum of R10$_{prot.}$. ^{1}H-NMR (400 MHz, DMSO-d6) δ 8.61 – 8.36 (m, 4H), 8.32 – 7.91 (m, 7H), 7.75 – 6.25 (m, 30H), 4.57 – 4.08 (m, 8H), 3.84 (s, 1H), 3.73 (s, 1H), 3.17 – 3.05 (m, 20H), 3.05 – 2.96 (m, 20H), 2.55 (s, 30H), 2.49 (s, 30H), 2.14 – 1.95 (m, 30H), 1.81 – 1.29 (m, 100H).

2.5.3 *Mass spectrometry*

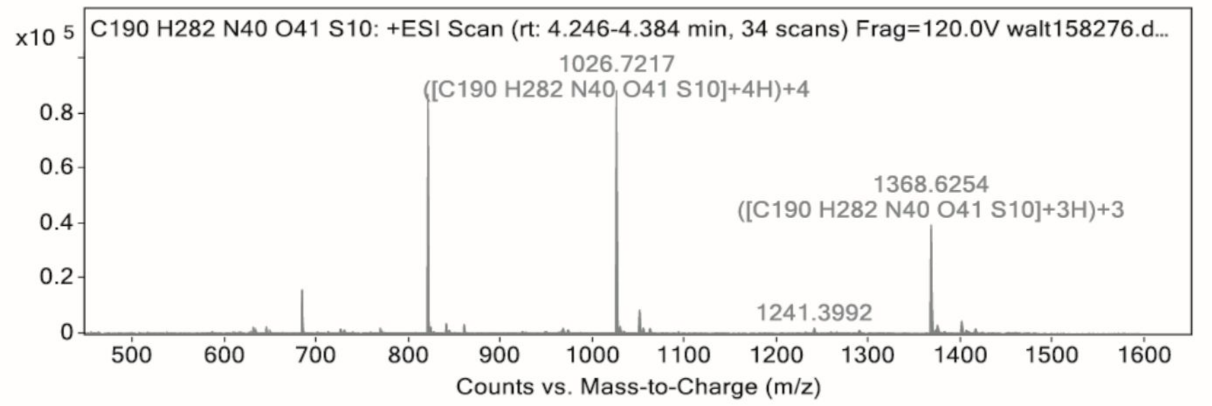

Figure S19. Mass spectrum of R10$_{prot.}$. HRMS: $(M+3H)^{+3}$ *m/z* calculated for $C_{190}H_{285}N_{40}O_{41}S_{10}^{3+}$: 1368.6237, found: 1369.6257.

2.5.4 HPLC

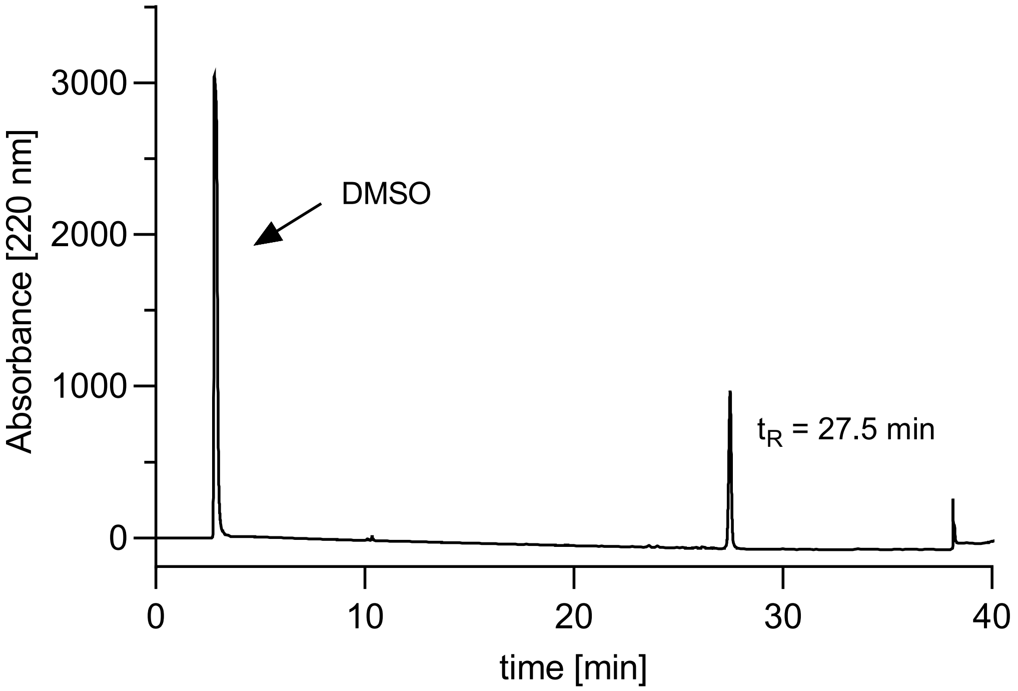

Figure S20. RP-HPLC analysis of R10$_{prot.}$. Retention time t_R of R10$_{prot.}$: 27.5 min.

3 Characterization of PLA-PEG Block Copolymers

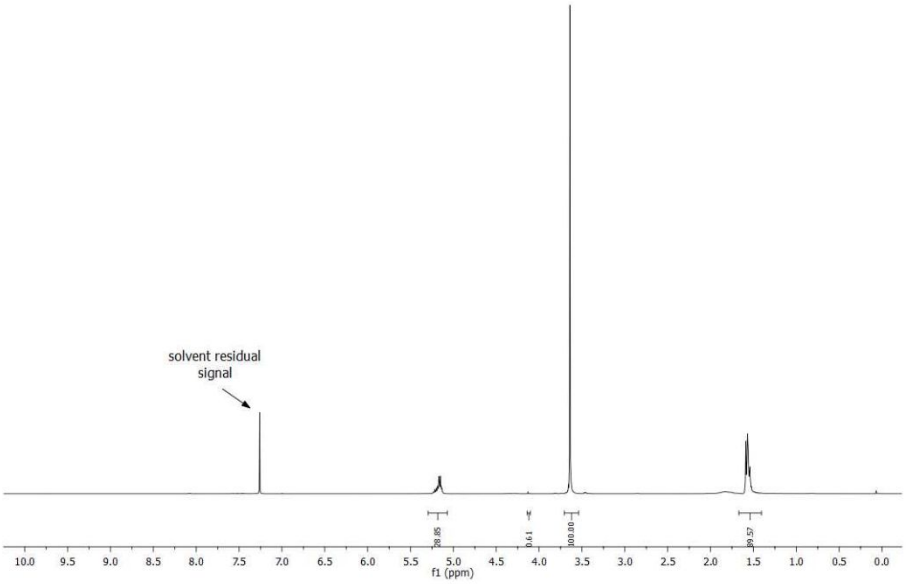

Figure S21. ^{1}H-NMR spectrum of COOH-PEG$_{5k}$PLA$_{10k}$. ^{1}H-NMR (400 MHz, $CDCl_3$) δ (ppm) 5.30 – 5.06 (m, 29H) (-(CH_3)H-); 4.13 (s, 1H) (-OCH_2CH_2-O(CO)-); 3.64 (s, 100H) (-OCH_2CH_2-); 1.60 – 1.37 (m, 90H) (-C(CH_3)H-). PEG (5000 Da) signal normalized to an integral area of 100. Integrating the PLA peaks resulted in a total molecular weight of the polymer of 14989 Da.

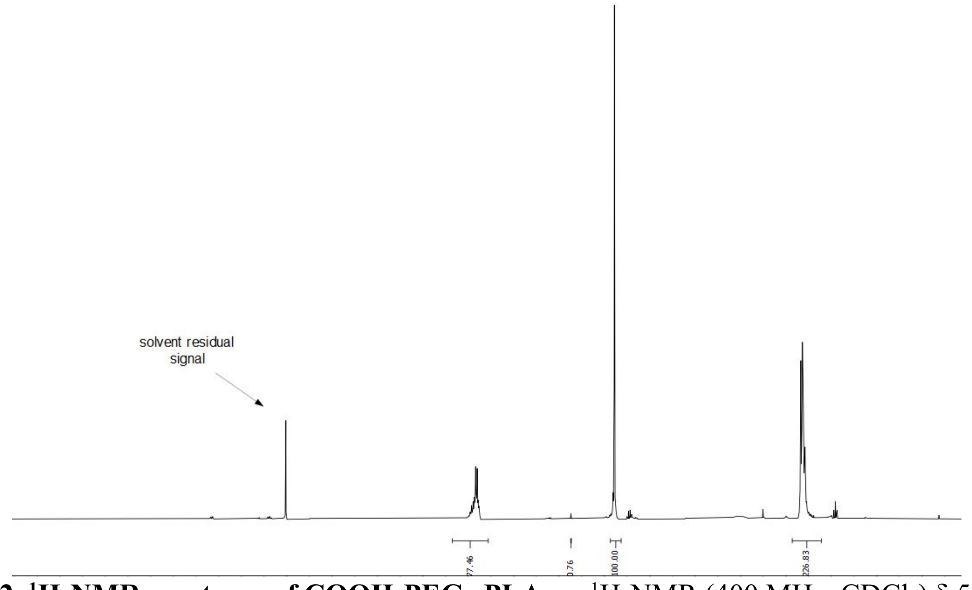

Figure S22. ^{1}H-NMR spectrum of COOH-PEG$_{2k}$PLA$_{10k}$. ^{1}H-NMR (400 MHz, $CDCl_3$) δ 5.40 – 5.02 (m, 77H) (-(CH_3)H-); 4.12 (s, 1H) (-OCH_2CH_2-O(CO)-); 3.64 (s, 100H) (-OCH_2CH_2-); 1.67 – 1.35 (m, 227H) (-C(CH_3)H-). PEG (2000 Da) signal normalized to an integral area of 100. Integrating the PLA peaks resulted in a total molecular weight of the polymer of 11983 Da.

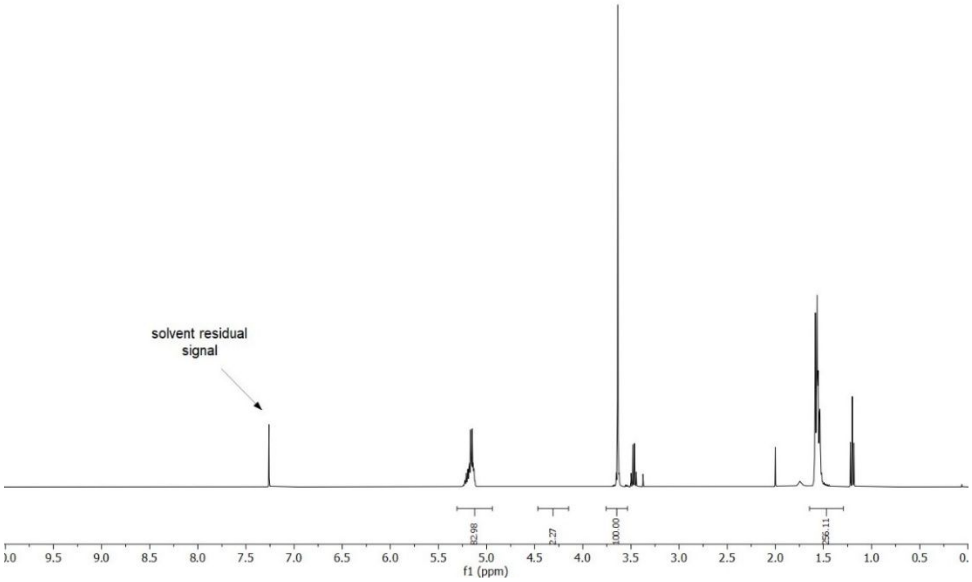

Figure S23. ^{1}H-NMR spectrum of MeO-PEG$_{2k}$PLA$_{10k}$. ^{1}H-NMR (400 MHz, $CDCl_3$) δ 5.31 – 4.93 (m, 33H) (-(CH_3)H-); 4.40 – 4.21 (m, 2H) (-OCH_2CH_2-O(CO)-); 3.64 (s, 100H) (-OCH_2CH_2-); 1.65 – 1.45 (m, 256H) (-C(CH_3)H-). PEG (2000 Da) signal normalized to an integral area of 100. Integrating the PLA peaks resulted in a total molecular weight of the polymer of 11524 Da.

4 Polymer Modification and Deprotection of Side Chain-Protected Amino Acids

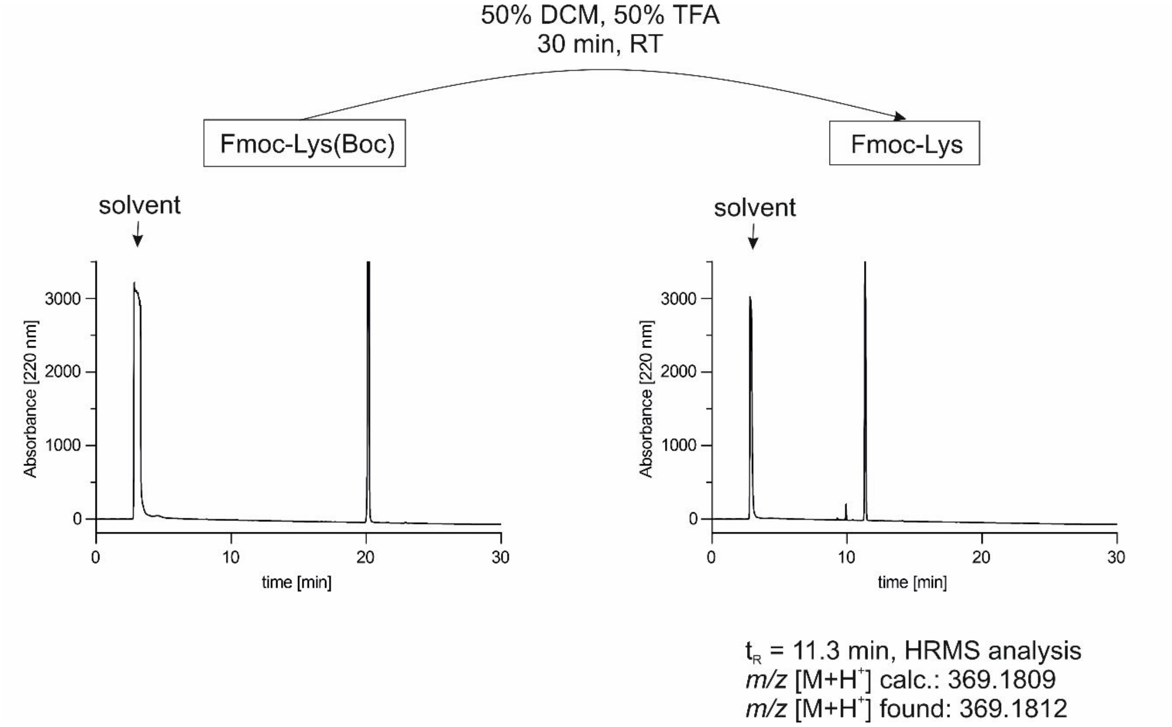

Figure S24. Test deprotection of Boc.

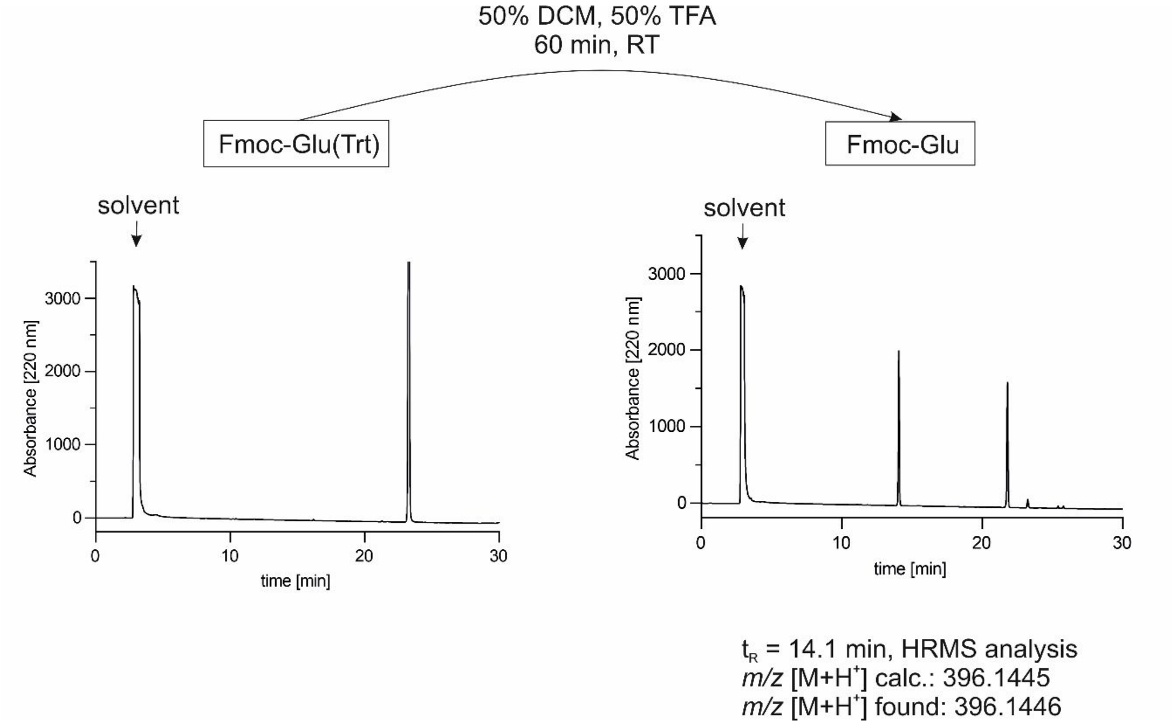

Figure S25. Test deprotection of Trt.

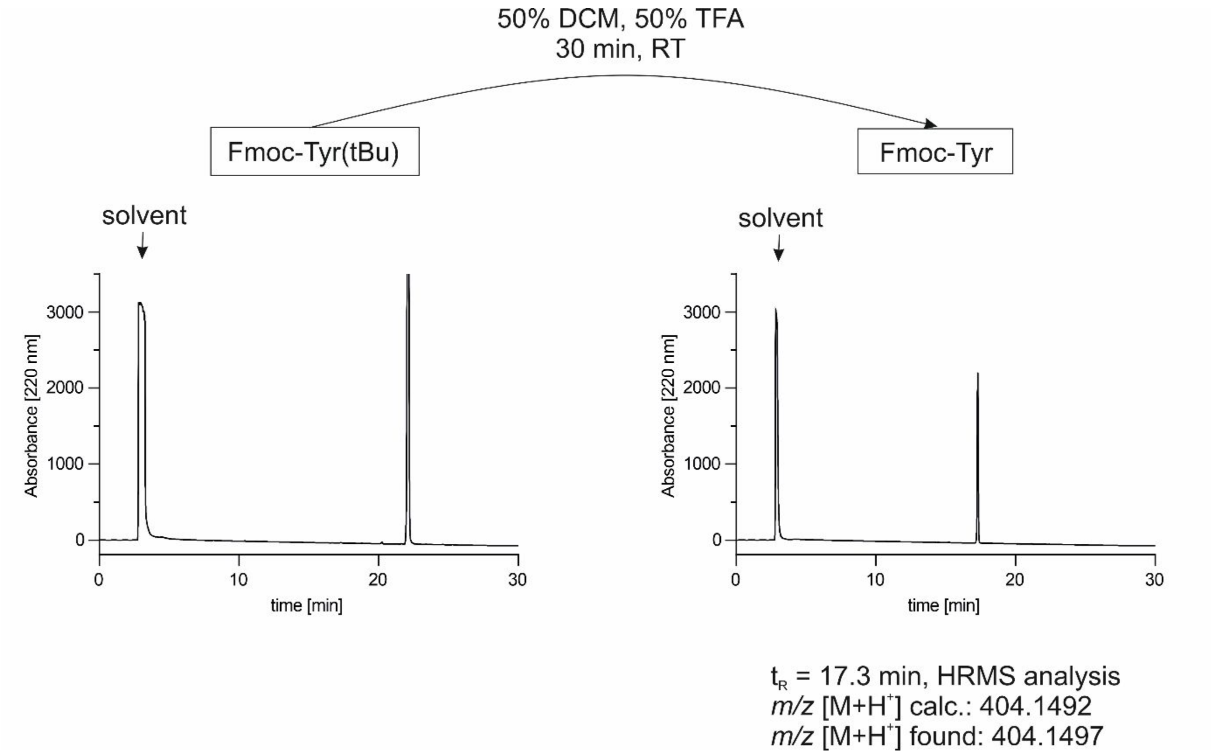

Figure S26. Test deprotection of tBu.

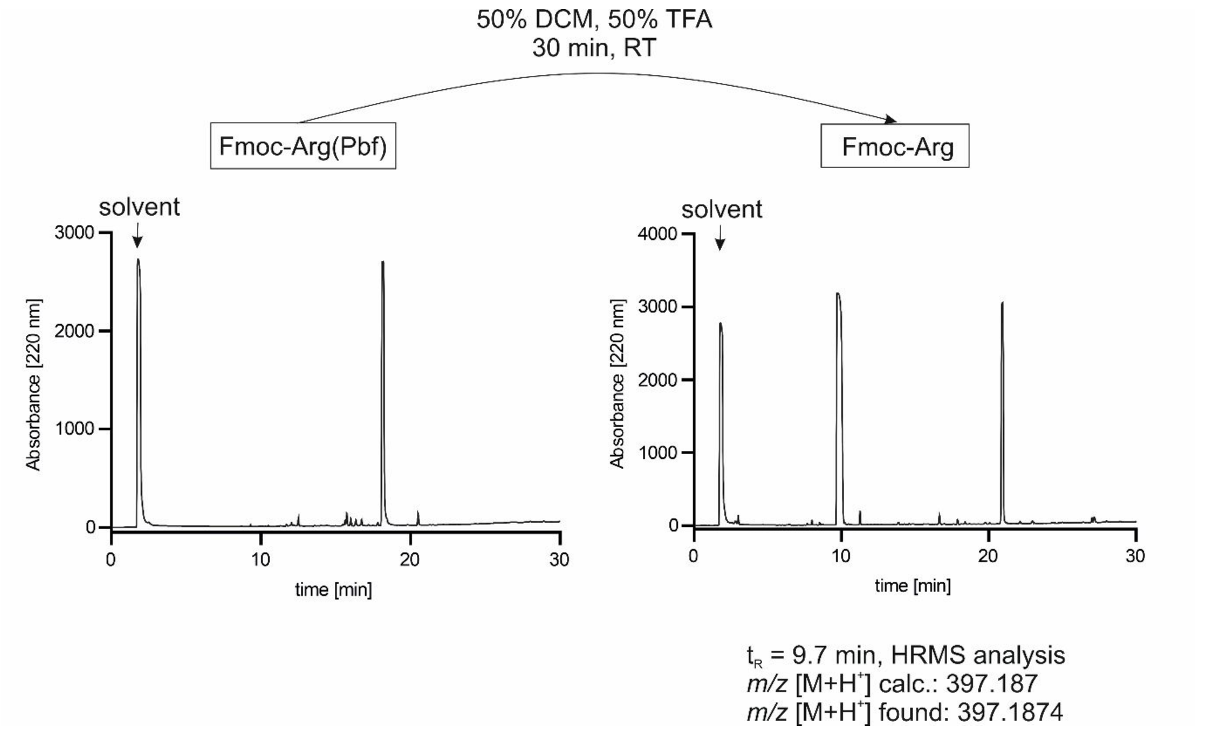

Figure S27. Test deprotection Pbf.

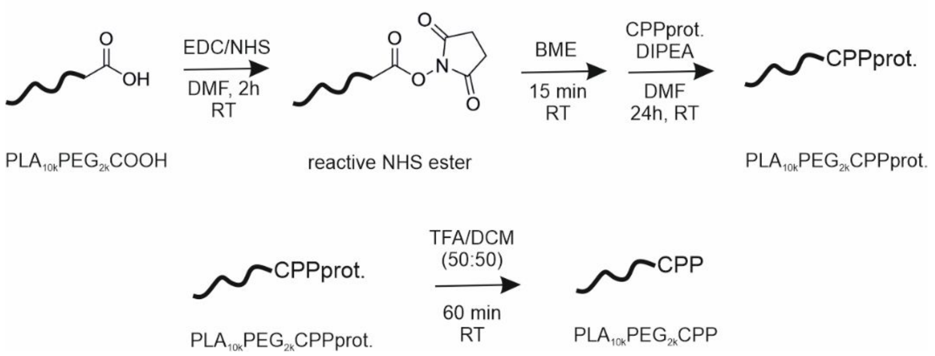

Scheme S2. Modification of the PLA10k-PEG2k-block copolymer used as shell component for particle preparation with different CPPs. Sidechain-protected amino acids were used within the CPP synthesis to allow subsequent specific coupling via the N-terminus ($PLA1_{0k}PEG_{2k}$CPPprot.). Afterwards, the coupled CPP was deprotected in a mixture of DCM and TFA (50/50), resulting in the free CPP attached to the polymer as reaction product ($PLA_{10k}PEG_{2k}$CPP).

5 Characterization of CPP-modified NPs

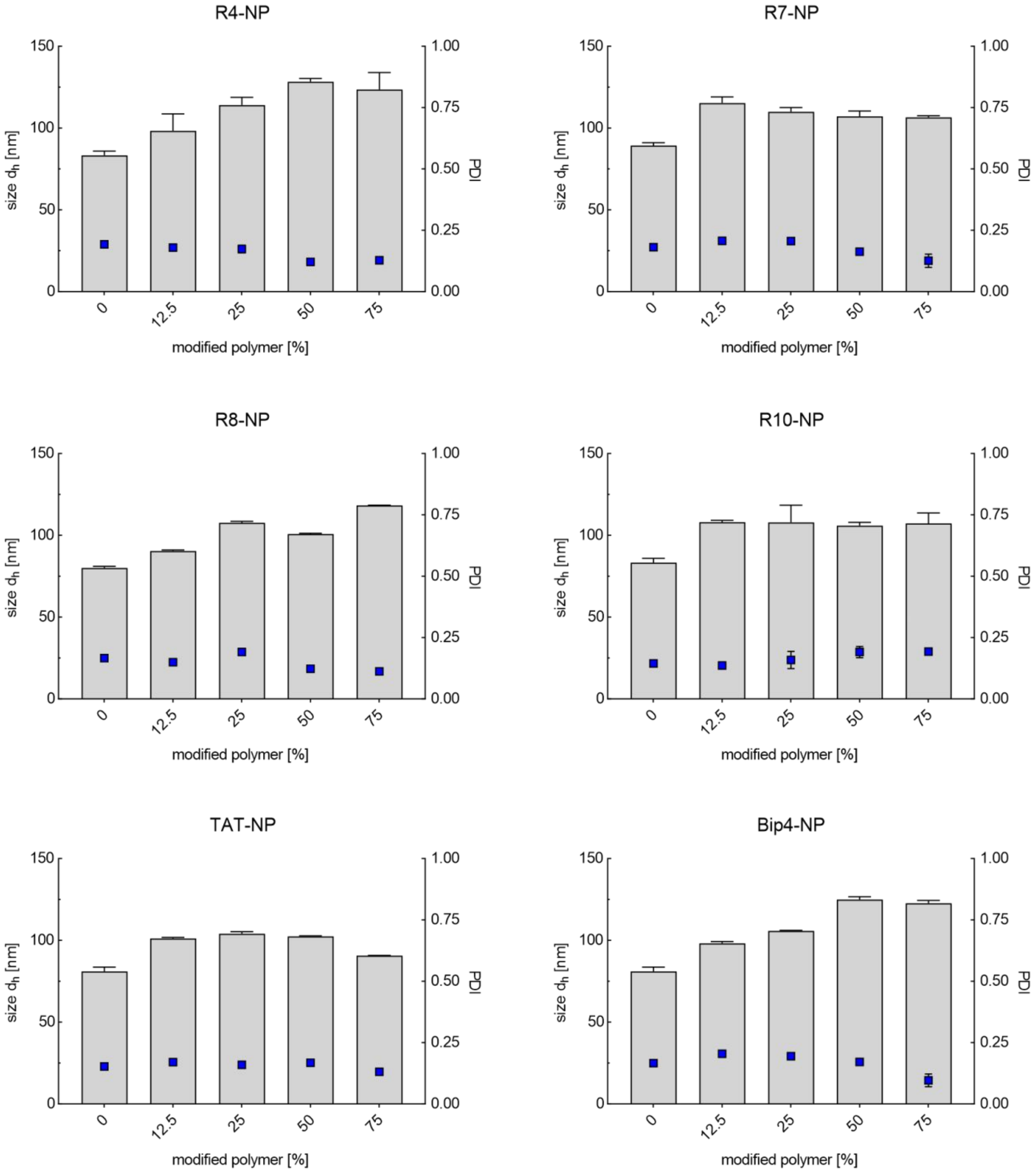

Figure S28. Characterization of size and PDI value of CPP-modified nanoparticles. The nanoparticle size was determined by nanoparticle tracking analysis (NTA) and shown as gray bar; the PDI value was analyzed via dynamic light scattering (DLS) and indicated as blue square. The particles consisted of core-polymer (PLGA) and shell-polymer chains of uniform length ($PLA_{10}kPEG_{2k}$). Therefore, the coupled cell-penetrating peptide (CPP) was localized on the nanoparticle surface. Different mass-ratios of CPP-modified polymer and unfunctionalized, uncharged methoxy polymer were evaluated as indicated in the diagrams. By modifying the particles with CPPs, the size increased for all ligands compared to unmodified control nanoparticles (n=3 technical replicates).

6 Uptake Behavior of CPP-modified Nanoparticles

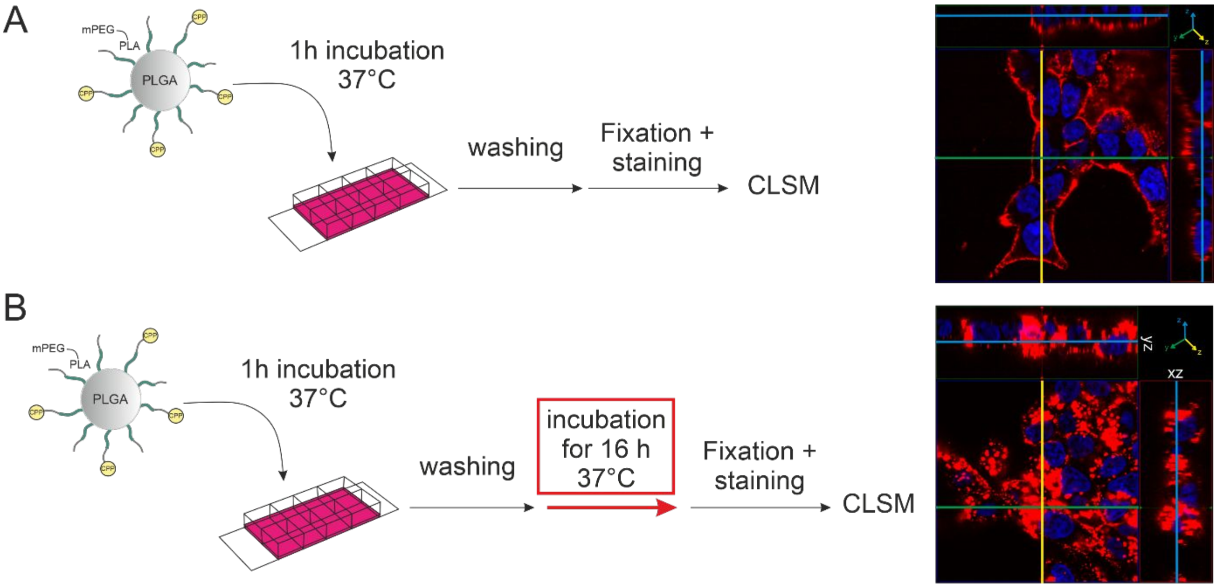

Figure S29. CLSM images of time dependence of R7-modified NP uptake into HEK293 cells. (A) Procedure ibidi-slide preparation and Z-stack of nanoparticle binding/uptake after 1h of incubation time. **(B)** Procedure ibidi-slide preparation and Z-stack of nanoparticle binding/uptake after 1h of incubation and additional 16 hours of incubation after nanoparticle expiration and washing. Nanoparticles were covalently core-labeled with Cy5 (red). Cell nuclei were stained with DAPI (blue). After 1 h of incubation, the majority of the nanoparticles were bound to the nanoparticle surface. After an additional incubation period of 16 hours, the nanoparticles were taken up into the cells.

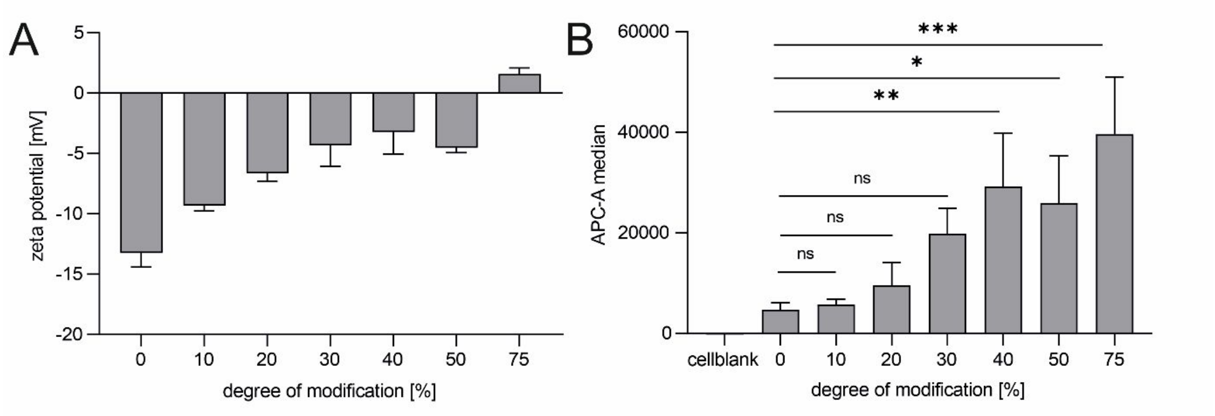

Figure S30. Shielding of R7 by $PLA_{10k}PEG_{5k}COOH$ polymer led to the loss of zeta-potential and significant uptake improvement-correlation in HEK293 cells. The cells were incubated with Cy5-labeled nanoparticles for 1 h at 37°C and afterwards analyzed via flow cytometry. **(A)** Zeta potential measurements of the particles. **(B)** Flow cytometric evaluation. Results represent mean ± SD (n = 3, levels of statistical significance are indicated as $^*p \leq 0.05$, $^{**}p \leq 0.01$, $^{***}p \leq 0.001$, $^{****}p \leq 0.0001$).

7 Cytotoxicity of Modified Nanoparticles

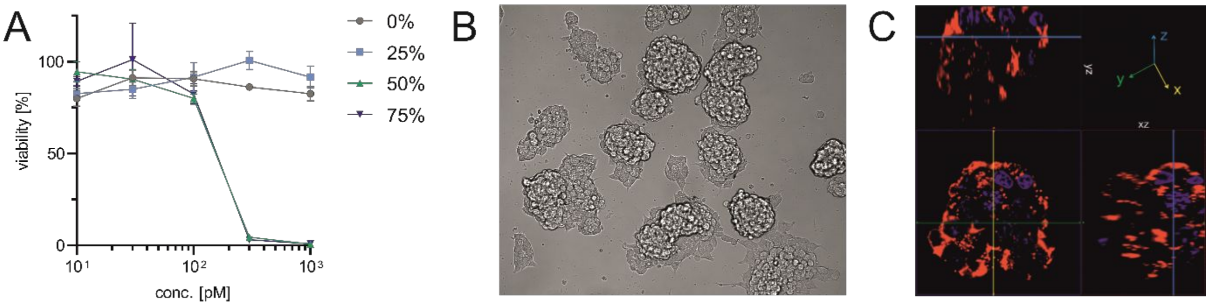

Figure S31. Evaluation of the cytotoxicity of R10-modified nanoparticles. (A) MTT assay performed with L929-cells. The different DOMs investigated are indicated in the figure legend. **(B)** TL-image showing the aggregation of HEK293 cells after treatment with R10-modified NPs DOM 50% in a concentration of 100 pM after 1 hours of incubation with particles and further 15 h of incubation after particle removement. **(C)** CLSM image: z-stack orthogonal view showing the aggregation of HEK293 cells in 3D.

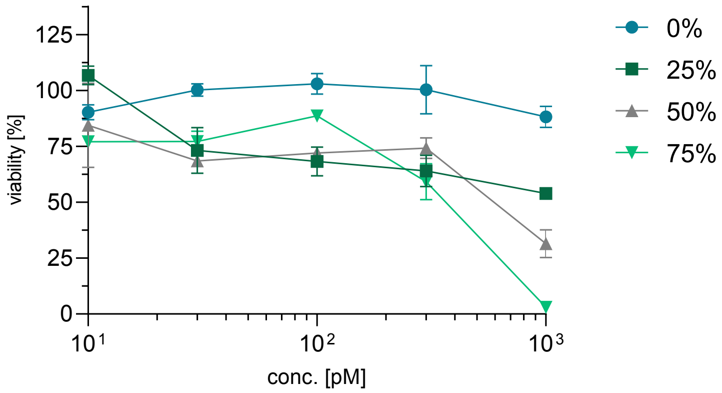

Figure S32. Effect of CPP shielding on R10-NP-cytotoxicity. The CPPs were sterically shielded by the usage of 25% longer polymers ($PLA_{10k}PEG_{5k}COOH$) and the cytotoxicity was evaluated via an MTT assay performed with L929-cells. The different DOMs investigated are indicated in the figure legend. Compared to particles with unshielded R10 a shift of cytotoxicity to higher nanoparticle concentrations could be detected.

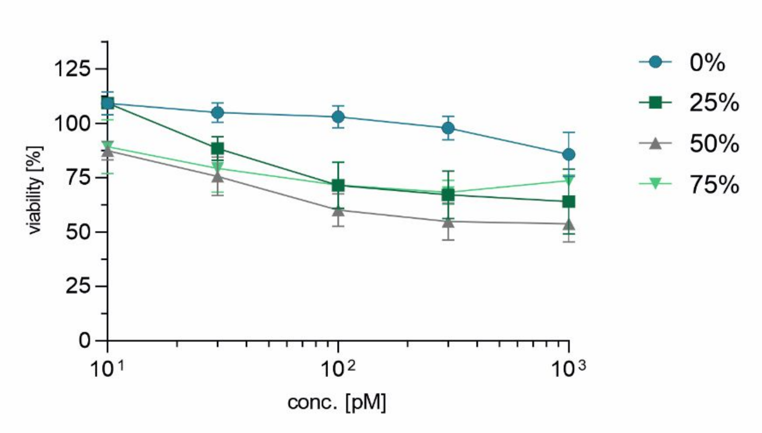

Figure S33. Evaluation of the cytotoxicity of NPs surface-modified with TAT(47-57). The MTT assay was performed with L929 cells. The different DOMs investigated are indicated in the figure legend.

The cytotoxicity of MLN-TAT NPs and control NPs was additionally investigated with HEK293T-ACE2 cells under the conditions of the flow cytometry experiments. Since HEK293 cells show only weak adherence, the utilized 96-well plates were initially coated using Collagen A (Biochrom GmbH, Berlin, Germany). Therefore, 1.25 mL collagen A 1 mg/mL was mixed with 11.25 mL PBS pH 2.4 (pH adjusted with 37% hydrochloric acid), and 250 µL/well was added. The solution was incubated for 1 h at 37 °C. Afterwards, the collagen was removed carefully. The wells were washed using 250 µL DMEM+10% FBS. Directly after the coating step, 75,000 cells/well were seeded into the coated plate and incubated for 24 hours at 37 °C. Unlabeled MLN-TAT NPs and control NPs were prepared according to chapter 2.4 and the particle concentration was adjusted to 100 pM with Leibovitz Medium. The cell medium was aspirated and the particles were added and incubated for one hour. Subsequently, the particle solution was aspirated and the cells were incubated with 200 µL MTT working reagent, prepared as described in chapter 2.6, for 3 hours at 37 °C. All the following steps were performed according to chapter 2.6. A cell viability of 99.86±5.48 % was demonstrated after treatment with MLN-TAT-NPs and 92.97±6.51% after treatment with control-NPs.

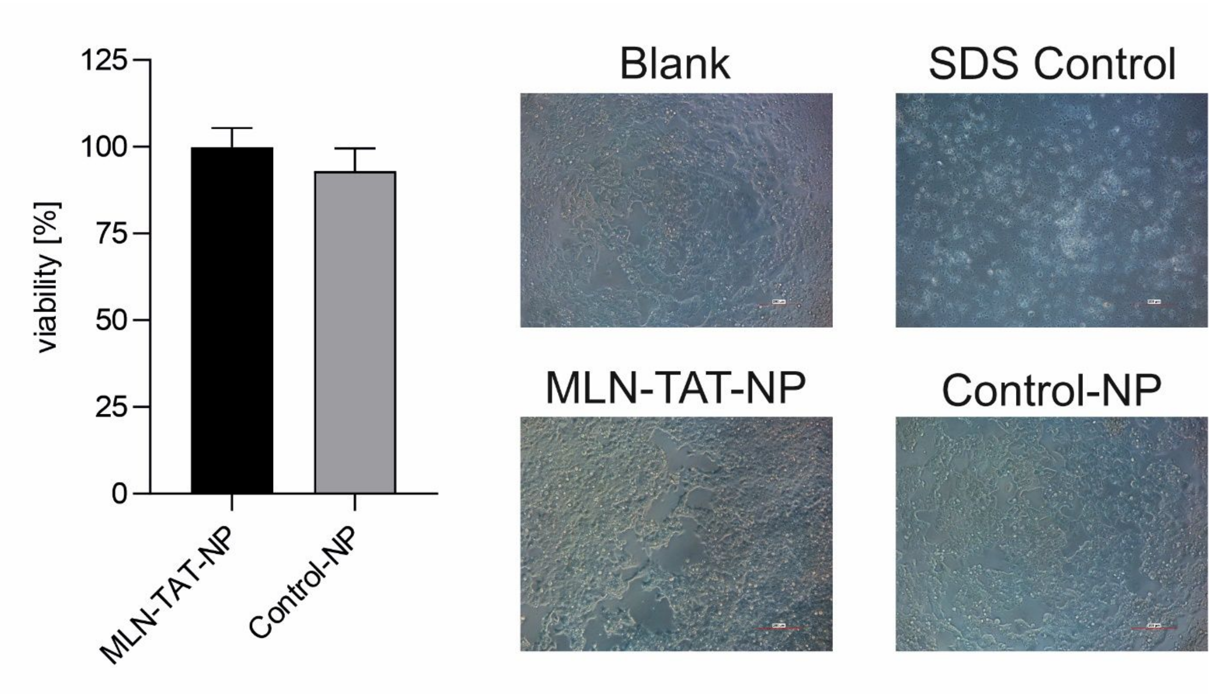

Figure S 35. Evaluation of the cell viability of HEK293T-ACE2 cells under flow cytometry experimental conditions. (n=6)
